# The *Janthinobacterium* sp. HH01 Genome Encodes a Homologue of the *V. cholerae* CqsA and *L. pneumophila* LqsA Autoinducer Synthases

**DOI:** 10.1371/journal.pone.0055045

**Published:** 2013-02-06

**Authors:** Claudia Hornung, Anja Poehlein, Frederike S. Haack, Martina Schmidt, Katja Dierking, Andrea Pohlen, Hinrich Schulenburg, Melanie Blokesch, Laure Plener, Kirsten Jung, Andreas Bonge, Ines Krohn-Molt, Christian Utpatel, Gabriele Timmermann, Eva Spieck, Andreas Pommerening-Röser, Edna Bode, Helge B. Bode, Rolf Daniel, Christel Schmeisser, Wolfgang R. Streit

**Affiliations:** 1 Abteilung für Mikrobiologie und Biotechnologie, Biozentrum Klein Flottbek, Universität Hamburg, Hamburg, Germany; 2 Laboratorium für Genomanalyse, Institut für Mikrobiologie und Genetik, Georg-August-Universität Göttingen, Göttingen, Germany; 3 Department of Evolutionary Ecology and Genetics, Christian-Albrechts Universität zu Kiel, Kiel, Germany; 4 Laboratory of Molecular Microbiology, Global Health Institute, School of Life Sciences, Ecole Polytechnique Fédérale de Lausanne (EPFL), Lausanne, Switzerland; 5 Center for integrated Protein Science Munich (CiPSM) at the Department of Biology I, Microbiology, Ludwig-Maximilians-Universität München, Martinsried, Germany; 6 Molekulare Biotechnologie, Institut für Molekulare Biowissenschaften, Goethe Universität Frankfurt, Frankfurt am Main, Germany; Belgian Nuclear Research Centre SCK/CEN, Belgium

## Abstract

Janthinobacteria commonly form biofilms on eukaryotic hosts and are known to synthesize antibacterial and antifungal compounds. *Janthinobacterium* sp. HH01 was recently isolated from an aquatic environment and its genome sequence was established. The genome consists of a single chromosome and reveals a size of 7.10 Mb, being the largest janthinobacterial genome so far known. Approximately 80% of the 5,980 coding sequences (CDSs) present in the HH01 genome could be assigned putative functions. The genome encodes a wealth of secretory functions and several large clusters for polyketide biosynthesis. HH01 also encodes a remarkable number of proteins involved in resistance to drugs or heavy metals. Interestingly, the genome of HH01 apparently lacks the N-acylhomoserine lactone (AHL)-dependent signaling system and the AI-2-dependent quorum sensing regulatory circuit. Instead it encodes a homologue of the *Legionella*- and *Vibrio*-like autoinducer (*lqsA/cqsA*) synthase gene which we designated *jqsA*. The *jqsA* gene is linked to a cognate sensor kinase (*jqsS*) which is flanked by the response regulator *jqsR*. Here we show that a *jqsA* deletion has strong impact on the violacein biosynthesis in *Janthinobacterium* sp. HH01 and that a *jqsA* deletion mutant can be functionally complemented with the *V. cholera*e *cqsA* and the *L. pneumophila lqsA* genes.

## Introduction

Janthinobacteria are Gram-negative, motile, aerobic bacteria that are commonly isolated from soil and aquatic samples. They are grouped within the family Oxalobacteraceae of the class Betaproteobacteria [1]–[7] and they produce a range of secondary metabolites such as violacein [8], a purple, water-insoluble pigment. Janthinobacteria are well known for their antifungal effects on frog skins. By forming biofilms on the frog skins and releasing violacein and perhaps other secondary metabolites they reduce the mortality and morbidity of their host animals significantly [9]. Furthermore it was reported that janthinobacteria affect the survival of nanoflagellates [6] and that already nanomolar amounts of violacein produced by the microbe inhibit protozoan feeding of marine biofilm bacteria [10].

It is noteworthy that up to date the genomes of only very few janthinobacteria are available. Only a single complete genome sequence has been established for *Janthinobacterium* sp. Marseille [11], even though the 4.9 Mb partial genome sequences of *Janthinobacterium* sp. strain PAMC 25724 has been announced very recently [12] and the 6.2 Mb genome sequence of strain GC3 is available as a permanent draft (DOE Joint Genome Institute). Finally the genome of the distantly related *Chromobacterium violaceum* has been published earlier [13]. Within the current publication we have established the 7.10 Mb genome sequence of a recently isolated *Janthinobacterium* sp. HH01 (from here on designated HH01). This is so far the largest known genome within the genus *Janthinobacterium*. HH01 was isolated from an aquatic source and produces violacein in stationary growth phase.

One of the striking features identified in the HH01 genome concerns the mechanism involved in cell-cell communication. Many Gram-negative bacteria employ a N-acylhomoserine lactone (AHL)-dependent quorum sensing (QS) regulatory circuit for intraspecies cell-cell communication. The corresponding AHL signaling molecules are synthesized through LuxI-like proteins [14], [15]. In addition a second but interspecies specific signaling circuit is commonly observed in Gram-negative microbes. This QS system has been designated autoinducer 2 (AI-2) signaling circuit. It depends on the synthesis of small furanone-like molecules. AI-2 is synthesized through LuxS-like proteins [14], [16]. Interestingly, HH01 lacks the synthesis genes for both of these signaling systems. However, it encodes a third type of autoinducer synthase genes previously only functionally characterized in *Legionella pneumophila* and *Vibrio cholerae*. The *Legionella*- and *Vibrio*-like autoinducer synthase genes *lqsA and cqsA* are involved in the biosynthesis of specific α-hydroxyketone signaling molecules termed LAI-1 and CAI-1 [17]–[19]. The *lqsA and cqsA* homologue identified in the HH01 genome was designated *jqsA*. In addition to its identification we report on the construction of a *jqsA* deletion mutant. Although, we have not identified the chemical structure of the novel janthinobacterial autoinducer, which we have designated JAI-1, we provide evidence that it is involved in regulation and expression of the violacein biosynthesis genes. We also show that the *jqsA* deletion mutant (HH02) can be functionally complemented with the *lqsA* and *cqsA* genes and therefore might be potentially useful for the detection of homologues from other Proteobacteria.

## Materials and Methods

### Bacterial Strains, Plasmids and Culture Conditions

Bacterial strains and plasmids used in the present work are listed in Table S1. Primers used are listed in Table S2. *Escherichia coli* was grown at 37°C in lysogenic broth (LB) medium [29] (1% peptone, 0.5% yeast extract, 1% NaCl) supplemented with appropriate antibiotics. HH01 was grown in R2A medium [30] (0.05% yeast extract, 0.05% tryptone, 0.05% casamino acids, 0.05% dextrose, 0.05% soluble starch, 0.03% sodium pyruvate, 1.7 mM K_2_HPO_4_, 0.2 mM MgSO_4_, final pH 7.2 adjusted with crystalline K_2_HPO_4_ or KH_2_PO_4_). *V. cholerae* strains were incubated statically within 24-well plates and biofilm formation was scored after 24 hours of growth using a standard crystal violet approach modified from reference [31]. Unless otherwise specified, media were supplemented with antibiotics, as required, at the following final concentrations: ampicillin, 100 µg/ml; chloramphenicol, 12.5 µg/ml; kanamycin, 25 µg/ml; and gentamycin 10 µg/ml. For metal resistance tests copper, iron and zinc were supplied as following final concentrations in liquid cultures, inoculated with a cell number of 1×10^7^ cells/ml and incubated for 24 hours: CuCl_2_, 500, 600 and 700 µM; FeCl_3_×6H_2_O 1200, 1300, 1400 and 1500 µM; and ZnCl_2_, 400, 500 and 750 µM. Agar plates employed for assaying exoenzyme activities contained 2% of the respective substrate.

### *Caenorhabditis elegans* Survival and Developmental Assays

All experiments were done using the *C. elegans* N2 strain, maintained on nematode growth media (NGM) at 20°C and fed on the *E. coli* strain OP50 [32]. Clean eggs and synchronized L4 larvae were obtained by bleaching as described in [32]. HH01 and the violacein-negative mutant HH5-1 (Table S1) were grown on R2A agar plates containing 25 µg/ml kanamycin at 23°C. 500 µl of bacterial overnight culture were spread onto 100-mm R2A agar plates and 90 µl onto 60-mm R2A agar plates. Plates were then incubated at 20°C for three days before use in the assays. *E. coli* were grown overnight in LB medium at 37°C and spread onto NGM plates. For the *C. elegans* developmental assay clean eggs were transferred onto 100-mm R2A agar plates with either HH01, the violacein biosynthesis mutant or onto NGM plates with *E. coli*, the standard laboratory food for *C. elegans*, as control. 6 replicate plates were assayed per bacterial strain. Development was monitored for 4 days at 20°C. Finally, for *C. elegans* survival assay 30 L4 larvae were picked onto each of 5 replicate 60-mm agar plates per bacterial strain and incubated at 20°C. Worms were scored as dead or alive by gentle prodding with a platinum wire and alive worms were transferred onto fresh plates every day. Data were analyzed using Kaplan-Meier statistics and survival curves were compared using the log-rank test. Due to multiple testing a Bonferroni correction of the *p*-value was made leading to a significance level of *p*≤0.016.

### Scanning and Transmission Electron Microscopy

For scanning electron microscopy cells were fixed in paraformaldehyde (1%) and glutaraldehyde (0.25%), dehydrated by ascending alcohol series and dried at the critical point with Balzers CPD 030 Critical Point Dryer (BAL-TEC, Schalksmühle Germany). After coating samples with gold using a sputter coater SCD 050 (BAL-TEC), scanning electron micrographs were taken with a LEO 1525 (Zeiss, Jena, Germany). For transmission electron microscopy cells were fixed in 2.0% (v/v) glutaraldehyde for 2 h and 1% (w/v) osmium tetroxide overnight. The embedding was performed according to Spurr [33]. Ultrathin sections were prepared with a diamond knife DiATOME ultra 45° (Diatome AG, Biel, Switzerland) on the ultramicrotome Ultracut E (Leica-Reichert-Jung, Nußloch, Germany) and were stained with 5% uranyl acetate and lead citrate. The sample examination was done with the transmission electron microscope Leo 906E (Zeiss, Jena, Germany) equipped with a CCD camera model 794.

To visualize flagella, a small volume (3 µl) of actively growing cells from a dense suspension was dropped on a copper grid, coated with Mowital polyvinylbutyral in 0.3% chloroform. Cells were allowed to attach for about 1 minute and excess liquid was removed by absorbent paper. The grid was placed with the upper side on a drop of uranyl acetate (2%) and dried after a few seconds. Pictures were taken with a transmission electron microscope as indicated above.

### Violacein Measurement

Violacein quantification was performed following a previously published protocol with minor modifications [34]. To measure the violacein amount of a freshly grown culture a 2 ml sample was centrifuged for 2 minutes at 13,000 rpm and resuspended in 0.4 ml H_2_O_dest_. After vortexing, the cells were lysed by adding 0.4 ml 10% sodium dodecyl sulfate and incubated at room temperature for 5 minutes. Violacein was quantitatively extracted from this cell lysate by adding 0.9 ml of water-saturated butanol, briefly mixing, and centrifugation at 13,000 rpm for 5 minutes in a microcentrifuge. 0.5 ml from the upper butanol phase containing the violacein was mixed with 0.5 ml water-saturated butanol, centrifuged again at 13,000 rpm for 5 min. The absorbance was measured at 585 nm in a SmartSpec^TM^Plus spectrophotometer (Bio-Rad Laboratories GmbH, Munich, Germany). For complementation tests with culture supernatants 10 ml of the supernatants were sterilized by filtration. 100 µl of this culture filtrate were added to 5 ml R2A media supplemented with ampicillin and 1% of growing HH02 cultures. For complementation tests with culture extracts 1 ml of sterile filtered supernatant was extracted using ethyl acetate (1∶1). 10 µl extract of a dilution series was added to an exponentially growing HH02 culture. Usually the synthesized violacein was quantified after an incubation period of 24–48 hours at 22°C. For violacein measurements with HH02 a positive control (i.e. HH01) was always included in the experiments. To minimize variations in violacein assays, R2A culture media were prepared with nutrients from the same batch of chemicals.

### Molecular Methods, Mutagenesis and Electroporation of HH01

HH01 gene cloning steps were carried out with standard methods [29]. Transformation of HH01 was conducted by electroporation: For this HH01 was grown in R2A medium overnight and then diluted in 100 ml sterile medium to an optical density (OD600 nm) of 0.1. Cells were grown to an OD600 of 0.6 at 22°C. For the electroporation cells were placed on ice for 30 minutes prior to centrifugation at 4,000 g at 4°C for 10 minutes. After this initial centrifugation step the cells were resuspended in 1 ml ice-cold sterile H_2_O_dest._, transferred to a pre-chilled microcentrifuge tube, washed three times with 1 ml ice-cold H_2_O_dest._ and resuspended in H_2_O_dest._ to a final concentration of 10^10^ cells/ml. The cells were mixed with 1 µl EZ-Tn5™<KAN-2>TnpTransposome™ and up to 1 µg of plasmid DNA, respectively and transferred to a pre-chilled 1 mm-electroporation cuvette (BIO-RAD, Gene Pulser Cuvette, *E. coli* Pulser Cuvette). The electroporation pulse was applied at 2.5 kV, 25 µF, 200 Ω using a Bio-Rad Gene PulserXcell, 165–2662 (Bio-Rad Laboratories GmbH, Munich, Germany). The electroporated cells were immediately mixed with 500 µl R2A medium, incubated for two hours at 22°C and spread on selective R2A agar plates.

In order to isolate violacein negative mutants of HH01 a transposon mutagenesis library was established by applying the EZ-Tn5™<KAN-2>Tnp Transposome™ Kit (Epicentre, Madison, Wisconsin, USA). The kit was used following the manufactureŕs instructions and 8,500 mutants were generated. A total of 50 white or cream colored mutants were selected and the insertion site of the transposon of 39 of these was determined following the recently published protocol [28]. By this a single *trpF* mutant, (HH5-1) was obtained which did not produce detectable amounts of violacein (Table S1).

For the construction of a knockout mutant in the *jqsA* gene a 2.25-kb *Bam*HI-*Eco*RI- fragment containing the partial *jqsA* gene and a gentamycin resistance cassette were cloned in the suicide vector pNPTS138-R6KT [27]. This construct (pNPTS138-*jqsA*::gm) was transformed into HH01 by electroporation. Single recombinant clones carrying this construct were selected on R2A medium containing kanamycin (25 µg/ml). To obtain double recombinant mutants bacteria were streaked on the same medium in the presence of 10% sucrose but lacking kanamycin. The correctness of the obtained *jqsA* mutant was verified by PCR and using different primer pairs flanking the *jqsA* gene. The obtained PCR fragments were sequenced to verify the correctness of the mutation.

### Extraction of JAI-1 and CAI-1 Autoinducer from Bacterial Cells

*E. coli* cells overexpressing either the *jqsA* or the *cqsA* gene in the vector pBBR1MCS-2 (Table S1) [25] were induced by the addition of 1 mM IPTG and grown overnight in LB medium. Cells were removed by centrifugation and the supernatant was extracted using ethyl acetate (1∶1). After concentration in a rotavapor (Buchi RE 111, Switzerland) the extract was resuspended in methanol. To test the effect of the autoinducer extract on violacein biosynthesis 10 µl extract of a dilution series was used to induce a 1% *Janthinobacterium* sp. HH01 culture. Violacein measurement was done after 24 h. As control, an *E. coli* strain with an empty pBBR1MCS-2 vector was used.

### Sequencing, Annotation and Bioinformatic Tools

DNA for 454 sequencing was isolated under standard conditions using the peqGOLD Bacterial DNA Kits (peqlab Biotechnology GmbH, Erlangen, Germany). The extracted DNA was used to generate 454-shotgun and paired-end libraries according to the manufactureŕs protocols (Roche 454, Branford, USA). Five and one, respectively medium lane of a Titanium picotiter plate was used for sequencing of the libraries, resulting in 1248653 total reads with 120,434 paired reads. The reads were *de novo* assembled using the Roche Newbler assembly software 2.3 (Roche 454). We created 7.3 Mb non-redundant sequences on 1,957 contigs with a size of 50 nt to 127,075 nt. Further a large insert-fosmid library was constructed according to the Copy Control fosmid library production kit manual (Epicentre Biotechnologies, Madison, WI, USA). A total of about 2,400 fosmid clones were generated. This equals a three-fold coverage of the HH01 genome. Ends of 672 recombinant fosmids were sequenced using ABI 3730xl automated DNA sequencers (Life Technologies, Darmstadt, Germany), processed with Phred, and assembled using Phrap. PCR-based techniques were used to close the remaining gaps using both genomic DNA and fosmid clones as templates. Further for gap closure DNA of selected fosmids was isolated using standard protocols and sheared for the construction of small insert plasmid libraries. These were constructed with the TOPO TA Cloning™ Kit (Invitrogen) using the pCR®4-TOPO® vector and previously published protocols [35].

All manual editing steps were performed using the GAP4 software package v4.5 and v4.6 [36]. Coding sequences (CDS) and open reading frames (ORFs) were predicted with YACOP [37] using the ORF-finders Glimmer, Critica and Z-curve. All predicted genes were manually curated based on GC frame plot analysis, the presence of ribosome-binding sites, and comparison to known protein-encoding sequences employing the Sanger Artemis tool v13 [38]. Functional annotation was initially performed with the ERGO software tool [39] and the IMG/ER (Integrated Microbial Genomes/Expert Review) system [40]. All CDS were manually curated and verified by comparison with the publicly available databases SwissProt, EMBL (InterProScan) GenBank, COG, and Prosite using the annotation software IMG/ER (https://img.jgi.doe.gov/cgi-bin/er/main.cgi). Gene products were classified into functional categories performing a BLAST search against the COG database [41]. Comparative analyses of different organisms was done as described previously [35] using a bidirectional BLAST algorithm, combined with a global sequence alignment based on the Needleman-Wunsch algorithm [42]. ORFs were assumed to be orthologs at a similarity higher than 30% and a BLAST e-value lower than 10e-21. Visualization of the chromosome and other DNA sequences was done by using DNA Plotter [43].

This whole-genome shotgun project has been deposited at DDBJ/EMBL/GenBank under the accession AMWD00000000 in two contigs. The version described in this paper is the first version, AMWD01000000.

## Results and Discussion

### Key Traits of *Janthinobacterium* sp. HH01 and Overall Organization of its Genome

**Figure 1:**
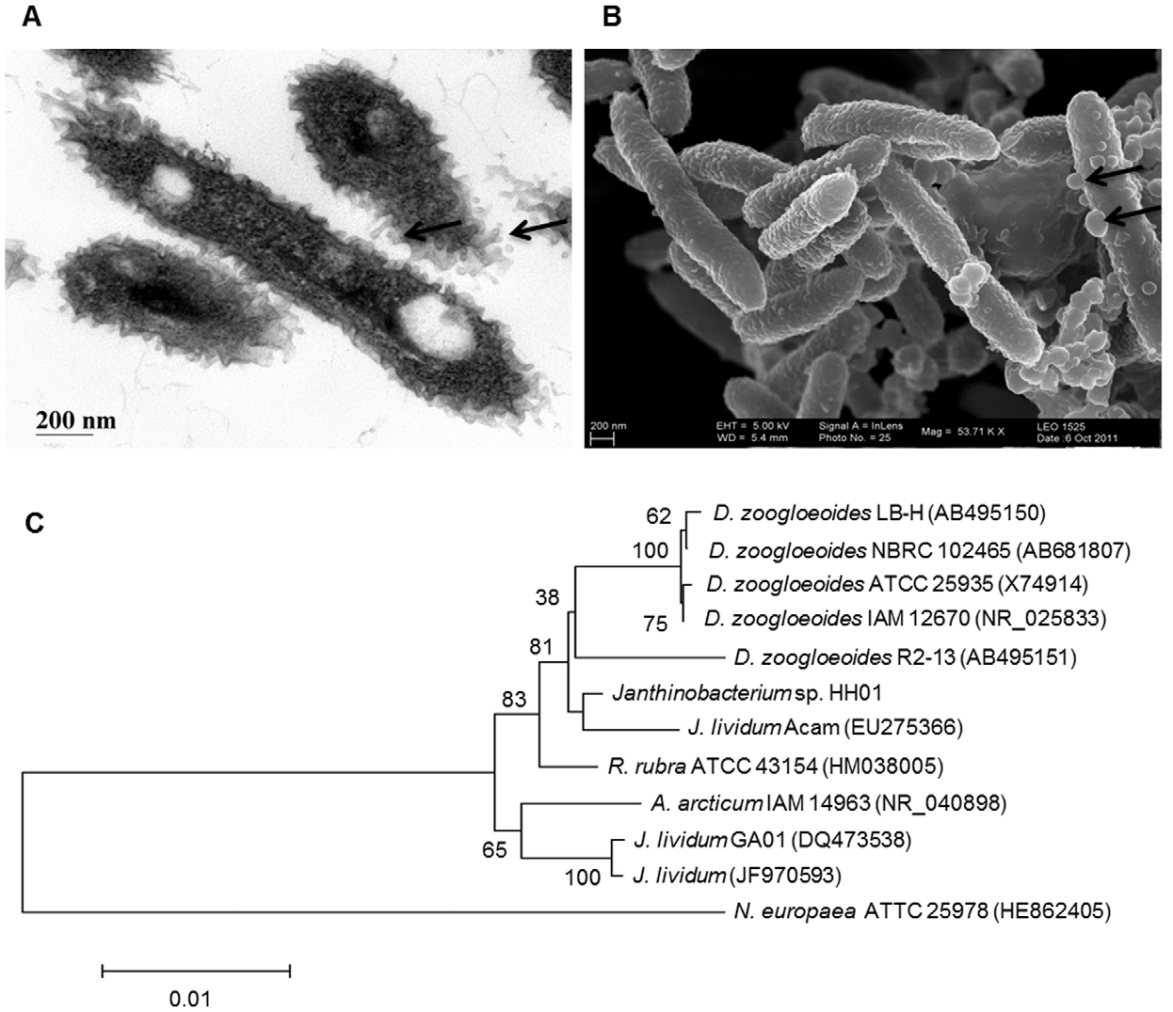
Transmission and scanning electron microscopic images of HH01 as well as a 16S rRNA-based tree. A) Transmission and B) scanning electron microscopic images of HH01. Arrows indicate observed vesicles on the HH01 outer cell surface. Scale bars of 200 nm are indicated in the images. C) 16S rRNA-based tree showing the phylogenetic affiliation of HH01. The tree was constructed using the neighbor-joining algorithm in MEGA5 [45]. Topology was evaluated by bootstrap analysis (1000 repeats, with *N. europae*a as an outgroup). Only sequences longer than 1450 nucleotides of representatives of the next relative (≥97% similarity) species validly described were included. Numbers in parenthesis indicate the corresponding GenBank entries. Bootstrap values are shown as percentages at the branch points. The scale bar represents the expected number of changes per nucleotide position.

HH01 was recently isolated from a watering can at the Loki-Schmidt botanical garden of the University of Hamburg. It grew well at temperatures ranging from 10° to 28°C and it revealed a doubling time of approximately 1.1 h at 22°C in R2A liquid cultures under aerobic conditions. Scanning (SEM) and Transmission (TEM) electron microscopic studies indicated that the cells were rod shaped with an average length of 2–4 µm (Figs. 1A & 1B).

The cell surface was covered with small outer membrane vesicle-like or bleb-like structures and their occurrence was highly reproducible in different cell preparations (Figs. 1A & 1B). They were not observed when we analysed images from the closely related *Duganella violaceinigra* (Table S1) and are therefore no preparation artefacts. However, we can only speculate about their possible role during biofilm formation, transport of DNA and or secondary metabolite export as suggested earlier [44].

The phylogenetic relationship of HH01 was established using the neighbor-joining algorithm in MEGA5 [45]. The 16S rRNA phylogenetic analysis suggested that the closest relative of HH01 was *Janthinobacterium lividum* strain EU275366. Therefore we decided to group HH01 within the genus *Janthinobacterium* (Fig. 1C).

**Figure 2:**
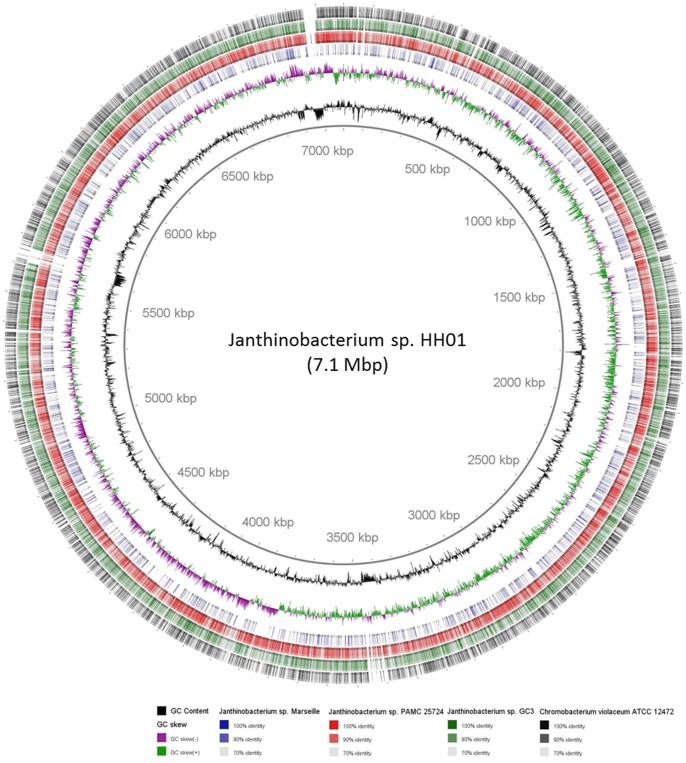
BlastP comparison of the *Janthinobacterium* sp. HH01 genome compared against genomes of closely related species. The innermost rings indicate the GC content (black) and GC skew (purple/green). The outer rings represent the genomes of the following microbes in different colorings: *Janthinobacterium* sp. Marseille, blue; *Janthinobacterium* sp. PAMC 25724, red; *Janthinobacterium* sp. GC3, green; and *C. violaceum* ATCC 12472, black. The genome map was created using BRIG (Blast Ring Image Generator; http://sourceforge.net/projects/brig) [46].

HH01 carries a single 7.10 Mb chromosome (Table 1, Fig. 2). Using pulse field gel electrophoresis no plasmids were detected (data not shown). The overall G+C content was 64.19% and the coding density was close to 92%. The bacterial chromosome encodes approximately 5,980 ORFs including a total of 84 tRNAs encoding all essential amino acids and 20 rRNA genes (Table 1) arranged in 7 rRNA clusters. Of the identified ORFs possible functions could be assigned to almost 80% of all predicted ORFs (Table 1 and Table S3).

**Table 1:** General features of the HH01 chromosome and closely related microorganisms.

	*Janthinobacterium* sp.				*C. violaceum*
Characteristics	HH01	GC3	PAMC25724	Marseille	ATCC12472
Size (Mbp)	7.10	6.26	4.98	4.11	4.75
G+C content (%)	64.19	65.54	60.60	54.23	64.83
rRNA genes	20	15	21	6	25
tRNA genes	84	81	80	46	98
Other RNA genes	12	16	13	14	20
Protein codinggenes	5,980	5,352	4,432	3,697	4,407
Functions assigned	4,877	4,365	3,538	2,510	2,687

The genome of *C. violaceum* ATCC12472 was derived from reference [13]; the genome information on *Janthinobacterium* sp. PAMC 25724 was obtained from reference [12] the genome information on *Janthinobacterium* sp. Marseille was derived from [11]; additional information and the genome information for *Janthinobacterium* sp. GC3 was extracted from the permanent and unpublished draft available at http://www.jgi.doe.gov/and using the IMG software at https://img.jgi.doe.gov/cgi-bin/er/main.cgi.

The likely origin of replication [47] was identified based on G+C skew, the position of chromosomal replication initiator protein (DnaA, Jab_2c34420), the DNA polymerase III beta subunit (DnaN, Jab_2c34430) and the DNA gyrase B subunit (GyrB, Jab_2c34440). The further analysis of the genome suggested that the HH01 genome differs significantly from the genomes of closely related species: *Janthinobacterium* sp. Marseille, *Janthinobacterium* sp. PAMC 25724, and between strain GC3. For instance the overall genome size with 7.10 Mb was significantly larger than any of the above mentioned partial or complete genomes (Table 1, Fig. 2). Also its G+C content with 64.19% is significantly different to the G+C content of *Janthinobacterium* sp. PAMC 25724 and *Janthinobacterium* sp. Marseille**.** However, the careful comparison of the genome of HH01 with the genomes related species suggested that larger syntenic regions occur between its genome and the genomes of *Janthinobacterium* sp. Marseille, PAMC 25724 and GC3 (Fig. 2). The overall genome organization is summarized in Table 1 in comparison to closely related species.

### Metabolic and Catabolic Traits of HH01, Aerobic- and Anaerobic-respiration

A careful analysis of the genome data suggest that the microbe is probably capable to grow on a wide variety of different carbon and energy sources under aerobic and anaerobic conditions (Table S3). For carbon metabolism it uses most likely the Embden-Meyerhof-Parnas pathway for the degradation of glucose or other C6 carbon compounds; but also genes were identified to allow growth on C3 and C4 carbon compounds. All essential and required genes for the Embden-Meyerhof-Parnas and citric acid cycle were identified; and a number of sugar kinases were observed that are involved in the activation of the respective carbon compounds. Under anaerobic conditions it appears to be capable to use nitrate as electron acceptor. All the essential subunits of the respiratory nitrate reductase were identified in a larger cluster together with possible nitrate and nitrite transporter genes (Jab_2c31470-Jab_2c29100). HH01 encodes a large cluster of genes essential to the degradation of phenylacetic acid (Jab_2c29740-Jab_2c29870). It also appears to be capable to use larger polymers as carbon and energy source (i.e. chitin and starch). With respect to the degradation of both these polymers at least four potential chitinase genes (Jab_1c17120, Jab_1c17130, Jab_2c26460 and Jab_2c26470) and four amylolytic enzymes are encoded by the HH01 genome (Jab_1c09720, Jab_2c03620, Jab_2c25890 and Jab_2c25970). Additionally the genome encodes for a large number (>20) of possible proteases, lipases and esterases. Activity assays in the laboratory confirmed that HH01 produces and secrets amylases, proteases and lipases into the surrounding medium (data not shown).

Furthermore the genome encodes a complete set of genes needed for the degradation of urea (Jab_2c01140-Jab_2c01210). It also most likely produces and utilizes poly-beta-hydroxybutyrate as carbon storage compound. The required genes linked to the biosynthesis (*phbC*_1_, Jab_2c02890 and *phbC*_2_, Jab_2c10560) and the cognate regulator *phaR* (Jab_2c02880) upstream of *phbC_1_* were identified. In addition several loci involved in depolymerisation (Jab_2c22480, Jab_1c02790, Jab_1c02800) and utilization were identified (*bdhA_1_,* Jab_2c12470; *bdhA_2_*, Jab_1c22740 and *phaZ*, Jab_2c16360). Altogether the wealth of metabolic and catabolic genes makes the microbe an interesting resource for the mining of biocatalysts. It also suggests that the microbe quickly adapts to changing nutritional conditions in its natural habitat.

### Resistance Mechanisms

HH01 encodes a remarkable number of proteins involved in resistance to drugs or heavy metals (Table S4). Altogether more than 2.4% of its genome is devoted to genes linked to resistance mechanisms. HH01 encoded at least nine possible beta-lactamases, three tetracycline resistance proteins ‘class A’ (Jab_1c02970, Jab_1c09990, and Jab_2c01640) and a significant number of multidrug resistance proteins linked to the export of drug molecules. Laboratory experiments confirmed the resistance to a wide range of antibiotics (Table S4). However, HH01 is sensitive to treatment with the amino-glycoside antibiotics kanamycin, neomycin and gentamicin. Additionally a gene loci linked to the detoxification of chloramphenicol was observed (Jab_2c08860). Within this framework, we also observed a number of genes linked to the resistance towards heavy metals. Interestingly, several possible chromate, arsenate and telluride transporters were identified and appeared to be located in a larger cluster of genes ranging from Jab_2c03840-Jab_2c04220 encoding a total of 38 genes/ORFs. In our hands the microorganism was able to grow well in the presence of elevated levels of CuCl_2_ (0.6 mM), ZnCl_2_ (0.5 mM) and FeCl_3_ (1.3 mM). These findings suggest that the microorganism might be able to survive under harsh environmental conditions with respect to heavy metal occurrence.

### HH01 Cell Appendages and Motility

**Figure 3:**
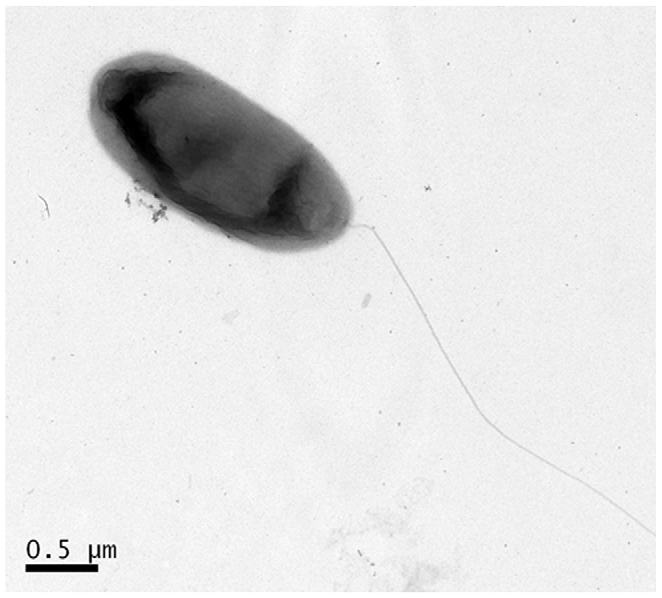
TEM image of HH01. A single flagellum attached to its cell pole is visible. Active cells were stained by uranyl acetate.

A surprisingly large number of more than 190 genes and ORFs (≈3%) are linked to assembly and function of flagellum- or pilus-like structures (Table S5). More than 80 genes are coding for regulatory or sensory functions linked to chemo- or aerotaxis. Many of the flagellum related ORFs are located in two larger but distinct clusters on the bacterial chromosome. One of these clusters encompassed at least 42 ORFs (Jab_2c12550-Jab_2c12970) and a second cluster encodes for a similar number of ORFs (Jab_2c23710- Jab_2c24150). HH01 is motile by flagella and using TEM, we were able to verify the presence of at least one subpolar or polar attached flagellum (Fig. 3). Flagella play a pivotal role for surface attachment and colonization [48], [98]. Thus a functional flagellum is certainly an important contribution to the ability of HH01 to cope with constantly changing environmental conditions and to effectively colonize surfaces.

Among other interesting features we identified a cluster of genes most likely involved in the assembly of a type 4 pilus (T4P). T4P are mainly involved in twitching motility, biofilm formation and host cell-interactions [50]. For the assembly of T4P more than 40 genes are known to be involved and some of them share homologies to components from the type 2 secretion system (T2SS) [50]–[52]. In HH01 the core pilus genes are located in two distinct clusters of genes encoding the *pilMNOPQ* (Jab_1c07780-Jab_1c07820); and the *pilVWXYE and fimT* genes (Jab_1c21010-Jab_1c21060) (Table S5). Interspersed throughout the genome many other genes possibly involved in the assembly of type 4 pili were observed. It is notable that T4P are involved in DNA uptake and the assembly of a pseudopilus appears to be required for natural competence. DNA uptake pili are one subclass of T4 pili [53], [54]. The binding of the exogenous DNA is usually facilitated by the *comEA* locus [54]. In HH01 the corresponding homologue is encoded by *comA* (Jab_2c04310). Other possibly competence-associated loci that were identified include the ComL DNA binding protein (Jab_2c02840), the ATP binding protein ComM (Jab_1c10770) genes and a possible DNA internalization protein (Jab_2c03830). The presence of many components linked to the assembly of T4P and the observation of major competence genes might suggest that HH01 is probably capable to incorporate foreign DNA fragments from its environment.

### The HH01 Genome Encodes Multiple Secretion Systems

In the genome of HH01 more than 80 loci were identified that are potentially linked to protein secretion. Altogether we observed the essential genes of the twin arginine (TAT)-pathway, the Sec multimeric system as well as the genes linked to the type 1, type 2 and the type 6 secretion systems (T1SS, T2SS, T6SS). The identified genes and ORFs linked to secretion are listed in Table S6.

In HH01 the essential components of the TAT pathway (*tatA/E*, *tatB* and *tatC)* [55] were identified on a conserved cluster (Jab_1c00810-Jab_1c00830). In addition HH01 appears to encode all required genes to build up the Sec multimeric transport system [47]. The signal recognition particle (SRP) mediates membrane targeting of translating ribosomes displaying a signal-anchor sequence [56]. In HH01 *ftsY* (Jab_1c13340) encodes the possible signal recognition particle-docking protein and Jab_2c29130 encodes the possible Ffh protein. T1SS comprise of three proteins that transport targeted proteins across both bacterial membranes to the extracellular space [57] and several genes linked to T1SSs were identified (Table S6). T2SSs, which are broadly conserved in Gram-negative bacteria, translocate exo-proteins (e.g. cellulases, lipases, etc.) from the bacterial periplasm into the surrounding media and are encoded by a set of 12–16 proteins [58]. HH01 encodes more than 30 genes possibly associated with T2SSs. The majority of these is observed in two conserved clusters each coding for 11 genes essential to the T2SS (Table S6). Altogether the relative high number of T2SS-associated genes and their organization in two distinct clusters suggests that HH01 encodes two distinct sets of T2SSs. This is rather unusual compared to the other janthinobacterial genomes. While *Janthinobacterium* sp. Marseille lacks a T2SS about 10–12 of the essential T2SS associated genes were identified in the draft genomes of *Janthinobacterium* sp. strains GC3 and PAMC 25724.

Type VI secretion systems (T6SS) are composed of 12–25 genes and they are often involved in protein transport of effector proteins into the eukaryotic host cells. Although, they are clearly related to pathogenicity they are observed in a wide range of pathogenic as well as many non-pathogenic microbes [59], [60]. The respective genes in HH01 were located in one larger cluster comprising of at least 16 genes involved in T6SS assembly (Jab_2c19030- Jab_2c19160). In addition a somewhat smaller cluster of genes possibly linked to the T6SS assembly was identified (Jab_1c15640-Jab_1c15710). Homologues were also identified in *Janthinobacterium* sp. GC3 and PAMC 25724 but not in *Janthinobacterium* sp. Marseille. It is perhaps noteworthy, that in none of the sequenced janthinobacterial species a T3SS was identified. While HH01 however, appears to lack a T4SS, detailed blast searches identified T4SS in GC3 and PAMC 25724.

In summary these data suggest that HH01 is able to effectively secrete proteins but it lacks the typical pathogen-associated systems (i.e. T3SS and T4SS).

### Secondary Metabolite Gene Clusters in HH01

A secondary metabolite analysis using the AntiSMASH program [61] of the HH01 genome revealed the presence of several gene clusters linked to the biosynthesis of potentially interesting metabolites (Table S7). Two clusters were identified that are coding for phytoene synthases (Jab_2c05760 and Jab_2c05810). Additionally, seven gene clusters encoding non-ribosomal peptide synthetases (NRPS) and one gene cluster encoding a NRPS-polyketide synthase (PKS) hybrid were identified [62]. NRPS and NRPS/PKS hybrids are important classes of enzymes responsible for natural product biosynthesis and clinically used examples for compounds derived from these enzyme systems are the antibiotic daptomycin [63] and the anti-cancer compound epothilone [64].

**Figure 4:**
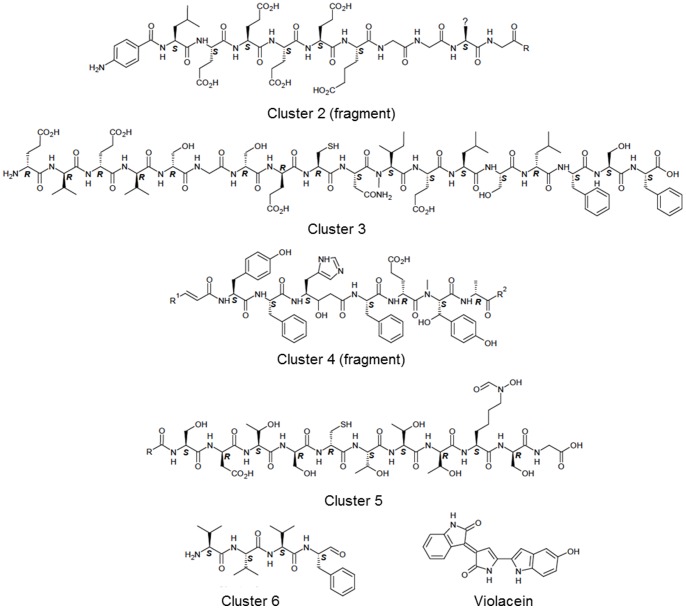
Predicted structures resulting from cluster 2–6. The predicted configuration is indicated by R- or S-nomenclature. All compounds are shown in the linear form but might be cyclic (for details see text). The HH01 genome was analyzed for secondary metabolite biosynthesis gene clusters using the AntiSMASH program [61]. Additionally, the genome was manually searched for genes encoding adenylation (A) and ketosynthase (KS) domains using a local BLAST server. All identified genes and/or gene clusters encoding the respective enzymes were then manually inspected and the predicted natural products resulting from the identified enzyme activities were drawn.

**Figure 5:**
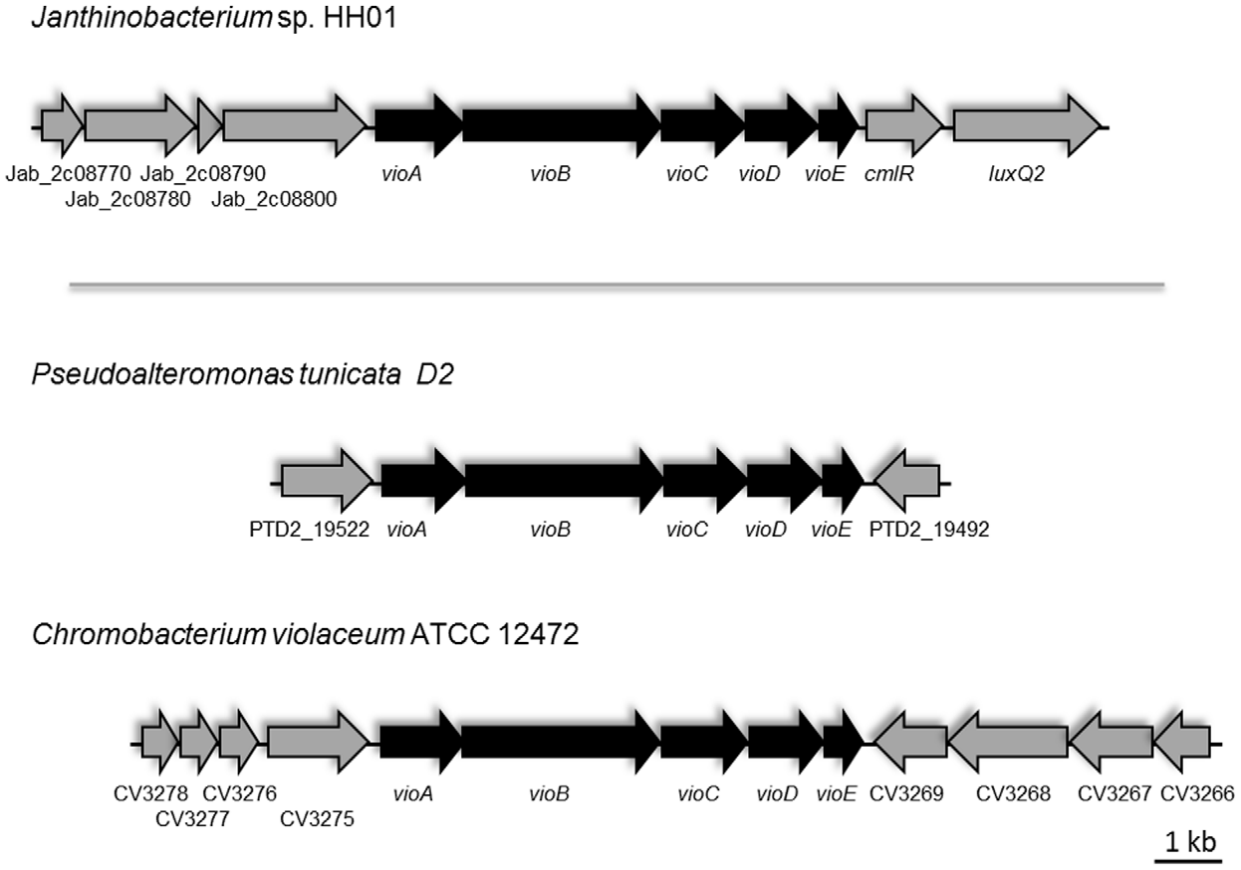
Violacein operon of HH01 and other violacein-producing bacteria. Conserved organization of the violacein biosynthesis operon of HH01 in comparison to violacein operons from *Pseudoalteromonas tunicata* strain D2 and *C. violaceum* ATCC 12472. Flanking genes not associated with the violacein biosynthesis are in grey; genes directly associated with violacein biosynthesis are in black. Arrows indicate direction of transcription. Jab_2c08770, two component regulator; Jab_2c08780, histidine sensor kinase, Jab_2c08790, FOG-domain containing hypothetical protein, Jab_2c08800, histidine sensor kinase; *cmlR*, potential chloramphenicol resistance protein; *luxQ2, luxQ* homologue; PTD2_19522, MATE efflux pump related protein; PTD2_19492 tryptophanyl t-RNA synthetase; CV3275, SpH family like protein; CV3276, hypothetical protein; CV3277, hypothetical protein; CV3278, cytochrome b561 protein; CV3266–C3269 hypothetical proteins. *P. tunicata* genes and ORFs were extracted from GenBank entry AAOH00000000 and the corresponding *C. violaceum* genes from GenBank entry NC_005085.1.

For the clusters 2–6 the structures were predicted using standard procedures [61] (Fig. 4). Compound 2 (from cluster 2) encodes a peptide with only L-amino acids, a high number of amino acids with carboxylic acid side chains and most likely a 4-aminobenzoyl starting unit. Compound 3 is an 18 amino acid peptide with almost exclusively D-amino acids at its N-terminus and L-amino acids at its C-terminus, respectively (Fig. 4). The compound from cluster 4 is very difficult to predict and might be a lipoheptapeptide with a central **γ**-amino acid resulting from a PKS module, which is part of the NRPS-PKS hybrid. Compound 5 might be a siderophore as a lysine monooxygenase and a formyltransferase are encoded within the cluster in addition to the NRPS, which would produce a lipoundecapeptide (11 amino acids) with a high number of serine and threonine moieties. Compound 6 is a tetrapeptide with a reduced C-terminus resulting from the C-terminal reduction (Red) domain of the NRPS. The predicted structures are summarized in Figure 4 in a linear form but it has to be mentioned that they can also be cyclic. Finally one cluster was observed coding for the synthesis of the purple pigment violacein (Fig. 4 and 5).

Altogether 2.0% of the HH01 genome is dedicated to secondary metabolism. This is significantly less compared to well-known secondary metabolite producers like *Streptomyces,* which encode 20–30 clusters and thereby devote 5–7% of their genomes to the synthesis of secondary metabolites [65], [66]. Nevertheless, due to the presence of unusual starting units (compound 2), N-methylation domains (compound 3 and 4) and use of unusual amino acids (compound 2, 4, and 5), HH01 might be an interesting model organism for secondary metabolite analysis. Surprisingly, cluster 3, 5, and 7 encode 4′-phosphopantetheinyl transferases (PPTase). It would be interesting whether these PPTase enzymes are specific for their individual clusters or can functionally complement each other, as usually a single PPTase is responsible for the activation of all NRPS and PKS enzymes [67]. Within this framework, the occurrence of two PPTases with the same function is already regarded as a rather unusual feature in a genome of a Gram-negative microorganism [68].

### HH01 Violacein Biosynthesis Affects *C. elegans* Survival and Nematode Development

The violacein biosynthesis genes identified in HH01 are located on a single and highly conserved operon comprising of the *vioABCDE* genes (Jab_2c08810-Jab_2c08850) (Fig. 5 and Table S7). In the 5′ direction of the *vioA* gene several histidine sensor kinases are encoded. Similar, in the 3′ direction of *vioE* a possible *luxQ* homologue is encoded (Jab_2c08870) (Fig. 5). It is noteworthy, that the closely related and sequenced janthinobacterial strains Marseille and GC3 did not appear to encode a functional violacein operon. Violacein is a bisindole and its biosynthesis has been observed in a wide range of different bacterial genera including *Janthinobacterium*, *Chromobacterium* [1], *Collimonas* [69], *Pseudomonas* [70], *Pseudoalteromonas* [71] and *Duganella* [72]. The violacein biosynthesis has been studied in the model organism *C. violaceum* in much detail [73]–[76]. The biosynthesis is managed through the gene products of the *vioABCDE* genes and by using tryptophan as a starting substrate [74], [75], [77]–[79]. In HH01 the violacein biosynthesis was most pronounced in late exponential growth phase and as expected from previous studies [80] the synthesis was stimulated by the addition of ampicillin (data not shown).

**Figure 6:**
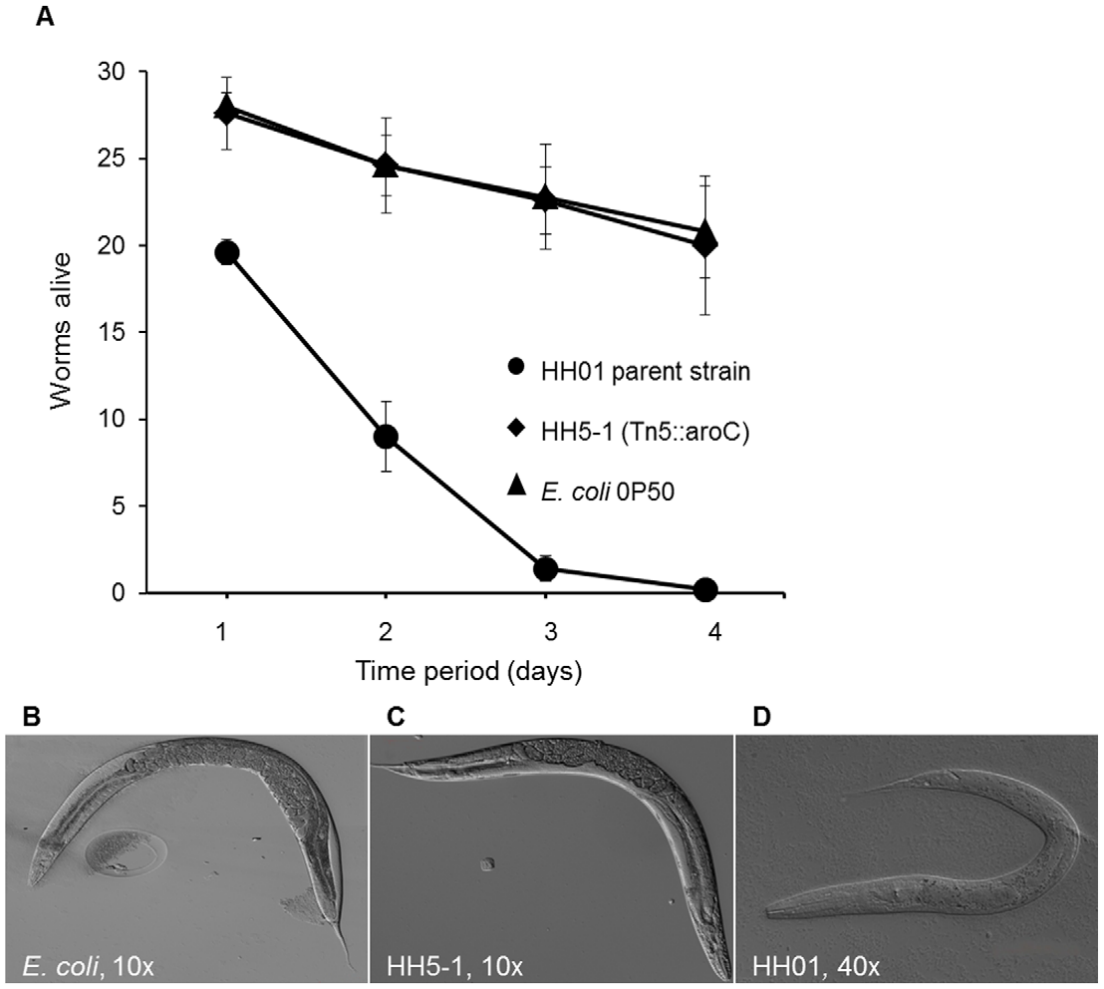
HH01 violacein biosynthesis affects *C. elegans* survival and nematode development. A) Decreased survival of *C. elegans* exposed to violacein-producing HH01. *C. elegans* grown on the violacein-producing parent strain HH01 died faster than worms on the *E. coli* control (p<0.001), while there was no significant difference in survival between worms grown on the violacein biosynthesis mutant and the *E. coli* control (p = 0.0375). B-D) Developmental arrest of *C. elegans* on violacein-producing janthinobacteria. B) DIC image (10x magnification) of a 4-day-old worm grown on *E. coli*. C) DIC image (10x magnification) of a 4-day-old worm grown on the violacein-negative mutant HH5-1. D) DIC image (40x magnification) of a 4-day-old worm grown on HH01. *C. elegans* grown on the violacein biosynthesis mutant and *E. coli* developed normally to the adult stage, whereas worms grown on the violacein-producing parent strain HH01 showed larval arrest.

Since it is well known that violacein has a wide range of biocidal effects [6], [81]–[86], we were interested to test if HH01 affects survival and development of *C. elegans*. In addition we wanted to elucidate, if possibly other factors or compounds produced by HH01 would influence *C. elegans* survival and development. In our tests the majority (>99%) of nematodes was dead after three days of exposure to HH01 (Fig. 6A). Furthermore our data suggested that HH01 has a strong influence on *C. elegans* development (Figs. 6B, 6C, 6D) as the nematodes did not develop over the larval stage in the presence of HH01 (Fig. 6D). In addition, we observed decreased locomotion and increased avoidance behavior (most worms were found outside the bacterial lawn) for the worms exposed to HH01 throughout the assays (data not shown). However, a violacein- and tryptophan-negative mutant (HH5-1, Table S1) did not affect *C. elegans* survival or development. *C. elegans* incubated with HH5-1 developed normally (Fig. 6C) and survived as well as in the presence of the *E. coli* control strain OP50 (Fig. 6B). Therefore it is highly likely that the violacein produced by HH01 itself or another compound derived from the tryptophan biosynthesis pathway was responsible for the observed phenotypes. This hypothesis was further supported by feeding *C. elegans* with *E. coli* cells carrying extra copies of the HH01 *vioA-E* genes (Fig. S1). Interestingly, Swem *et al.* [87] had observed that the toxicity towards *C. elegans* was not primarily caused by the purple pigment violacein produced by *C. violaceum*. Therefore, we speculate that in *C. violaceum* possibly several pathogen-related mechanisms are expressed that significantly affect *C. elegans* survival and which are not present in HH01 and/or which are not expressed in HH01 at the same level as in *C. violaceum*.

### HH01 does not Encode a N-acyl-homoserine-lactone (AHL) Regulatory Circuit

To analyze possible quorum sensing (QS) regulated circuits in this novel microbe detailed blast searches were performed. The HH01 genes and ORFs possibly linked to the synthesis and sensing of cell-cell communication signaling molecules are summarized in Table S8. AHLs (AI-1) are the key signaling molecules in the cell density-dependent system of gene regulation in many Gram-negative bacteria and they are usually synthesized through a LuxI like protein [15]. Employing the amino acid sequences of various LuxI homologues for blastP searches, we could not detect a homologue in the HH01 genome. Similarly *Janthinobacterium* sp. Marseille and PAMC 25724 appear to lack the AHL-dependent regulatory circuit (Table 2). It is however noteworthy that in the distantly related *C. violaceum* the synthesis of violacein is regulated through an AHL-dependent regulatory circuit [88]–[91].

**Table 2:** ORFs identified and involved in autoinducer biosynthesis in HH01 and closely related microorganisms.

	*Janthinobacterium* sp.				*C. violaceum*
Synthase type	HH01	GC3	PAMC 25724	Marseille	*ATCC 12472*
AHL	−	(+)	−	−	*+*
Ai-II	−	−	−	−	−
JAI-1	+	+	+	−	−

−, autoinducer synthase not detected, +, autoinducer synthase identified; (+), weak similarity observed to the known autoinducer I synthases from *C. violaceum* CviI and related species.

### HH01 Lacks a Gene Linked to the Synthesis of Autoinducer 2 (AI-2)

The AI-2 molecules are synthesized by a wide range of bacteria and are thought to be involved in interspecies cell-cell communication [16], [92]. They are composed of a furanone like ring structure [14] (and references herein) and their synthesis is accomplished through a S-ribosyl homocysteine lyase, (LuxS). Its receptor is the LuxPQ complex [93]. Interestingly, no homologue of the AI-2 synthase could be identified in our strain suggesting that HH01 most likely does not synthesize AI-2. Similarly, no such homologues were identified in any of the other janthinobacteria (Table 2). However, at least two potential sensor kinases for AI-2 molecules were identified in HH01. One of these (Jab_2c08870) was in proximity of the violacein biosynthesis genes (Fig. 5). Thus it is very well possible that HH01 senses AI-2 molecules.

### The HH01 Genome Encodes a Homologue of the *V. cholerae cqsA* and the *L. pneumophila lqsA* Gene

**Figure 7:**
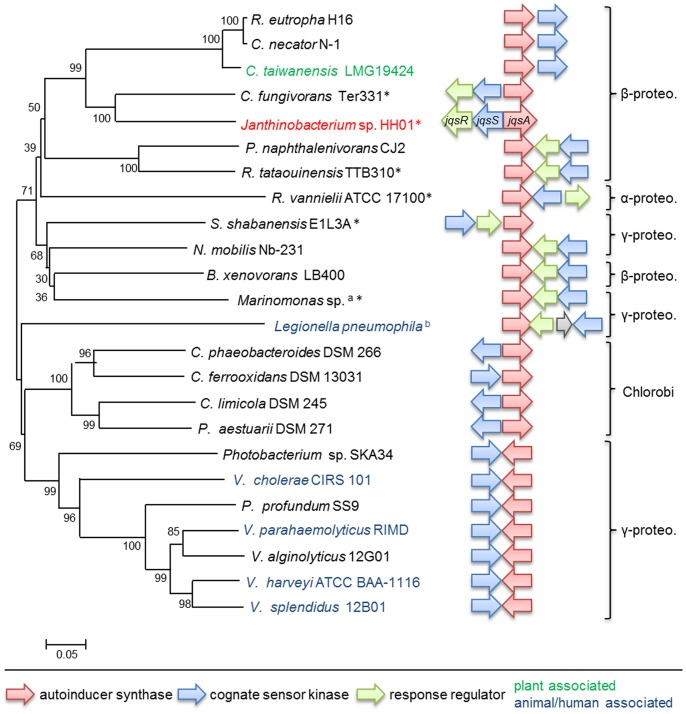
Phylogenetic analysis of *cqsA-, jqsA*- and *lqsA*-like autoinducer synthases in Gram-negative bacteria. The neighbor-joining phylogenetic analysis was performed using the MEGA5 software [45] version 5.1 and comparing amino acid sequences of the corresponding synthases. Homology searches for orthologous proteins were done in the IMG genome database in September 2012 with 3,938 completed or draft bacterial genomes present. The autoinducer synthase sequences of the following strains are included, numbers in parenthesis indicate the corresponding accession number: *R. eutropha* H16 (YP_728640), *C. necator* N-1 (YP_004680649), *C. taiwanensis* LMG19424 (YP_001796752), *C. fungivorans* Ter331 (YP_004750816), *P. naphthalenivorans* CJ2 (YP_983733*), R. tataouinensis* TTB310 (YP_004617950), *R. vannielii* ATCC 17100 (YP_004010985), *S. shabanensis* E1L3A (ZP_08550556), *N. mobilis* Nb-231 (ZP_01127067), *B. xenovorans* LB400 (YP_555293), *C. phaeobacteroides* DSM 266 (YP_912394), *C. ferrooxidans* DSM 13031 (ZP_01385258), *C. limicola* DSM 245 (YP_001942557), *P. aestuarii* DSM 271 (YP_002015366), *Photobacterium* sp. SKA34 (ZP_01162832), *V. cholerae* CIRS 101 (ZP_05420646), *P. profundum* SS9 (YP_133409), *V. parahaemolyticus* RIMD 2210633 (NP_800221), *V. alginolyticus* 12G01 (ZP_01260612), *V. harveyi* ATCC BAA-1116 (YP_001448208), *V. splendidus* 12B01 (ZP_00990208). a) *Marinomonas* sp. is summarized for: *M. mediterranea* MMB-1 (ATCC 700492); *M. posidonica* IVIA-Po-181. b) *L. pneumophila* is summarized for: Philadelphia-1 (YP_096734), Paris (YP_125092) and Lens (YP_127984). Bacterial genera that have previously not been reported [17] to contain a *cqsA/lqsA* homologue are marked with an asterisk.

Additional searches for possible autoinducer synthases identified a possible homologue of the *V. cholerae cqsA* and the *L. pneumophila lqsA* genes in HH01. In *V. cholerae* and *L. pneumophila* these genes are responsible for the synthesis of the CAI-1 and LAI-1 autoinducers, respectively [17] (and references herein). The corresponding HH01 gene (Jab_2c24330) was designated *jqsA.* It is adjacent with a two-component histidine sensor kinase designated *jqsS* (Jab_2c24340) and a two-component regulator gene designated *jqsR* (Jab_2c24350) (Fig. 7). JqsA reveals a 63% similarity (45% identity) to the corresponding *V. cholerae* El Tor strain N16961 protein and it revealed a 61% similarity (40% identity) with the *L. pneumophila* LqsA. However, the highest similarity of JqsA was observed to the homologue in the *Cupriavidus necator* strain N-1. JqsA is 79% similar (59% identical) to the orthologous protein in *C. necator*. A detailed phylogenetic analysis of the HH01 JqsA suggested that it grouped closely with orthologous proteins that were mostly derived from non-pathogenic environmental isolates (Fig. 7). A previous analysis by Tiaden and colleagues had already identified homologues of CqsA and LqsA in 10 different bacterial genera [17]. Surprisingly, only for the *Vibrio cqsA* and the *Legionella lqsA* gene a functional role has yet been defined [19], [94]. Therefore the identification of a JqsA/JqsS homologue in HH01 increases the diversity of known CAI-1/LAI-1-like signaling systems (Fig. 7). Since *Vibrio cholerae* lacks a LqsR homologue in its *cqsA/cqsS* gene cluster [17] (Fig.7) the presence of JqsR in the HH01 gene cluster might suggest that the janthinobacterial regulatory system is overall more closely related to the *Legionella*- than to the *Vibrio*-system.

In *V. cholerae cqsA* encodes a PLP-dependent aminoacyl-CoA transferase, that is responsible for the synthesis of CAI-1, a (*S*)-3-hydroxytridecan-4-one-like molecule. In *V. cholerae* CAI-1 is involved in the repression of virulence, in biofilm dissolution and it has been shown play a major role in natural competence [19], [21], [95]–[97]. Furthermore CAI-1 in *Vibrio harveyi* appears to be involved in the regulation of luminescence [93]. A recent study also suggested that the *V. cholerae* CAI-1 has impact on *P. aeruginosa* biofilm formation and thereby acts as an interspecies signaling molecule [98]. In Legionella a structurally related molecule, but carrying a longer carbon (C_15_) chain has been identified [18]. The LAI-1 signaling system in *L. pneumophila* promotes the pathogen-host cell interactions and it was shown to be involved in the regulation of extracellular filaments and a genomic island [94].

Interestingly, in all the microorganisms carrying a *cqsA*, *jqsA* or *lqsA-*like autoinducer synthase gene, the synthase gene is encoded adjacent to the cognate sensor kinase (Fig.7) [17]. The observation that the synthase is always linked to the corresponding sensor protein suggests that they might be acquired via horizontal gene transfer as part of a conserved gene cluster. It is also notable that in the sequenced partial genomes of *Janthinobacterium* sp. PAMC 25247 and GC3 a JqsA homologue was identified (Table 2). However, using blast analyses no homologue was identified in *Janthinobacterium* sp. Marseille (Table 2). These findings suggest that a *jqsA*-like gene and JAI-1-dependent regulatory circuit is perhaps not an essential element of the janthinobacterial core genome.

### Functional Verification of the HH01 *jqsA* Gene

**Figure 8:**
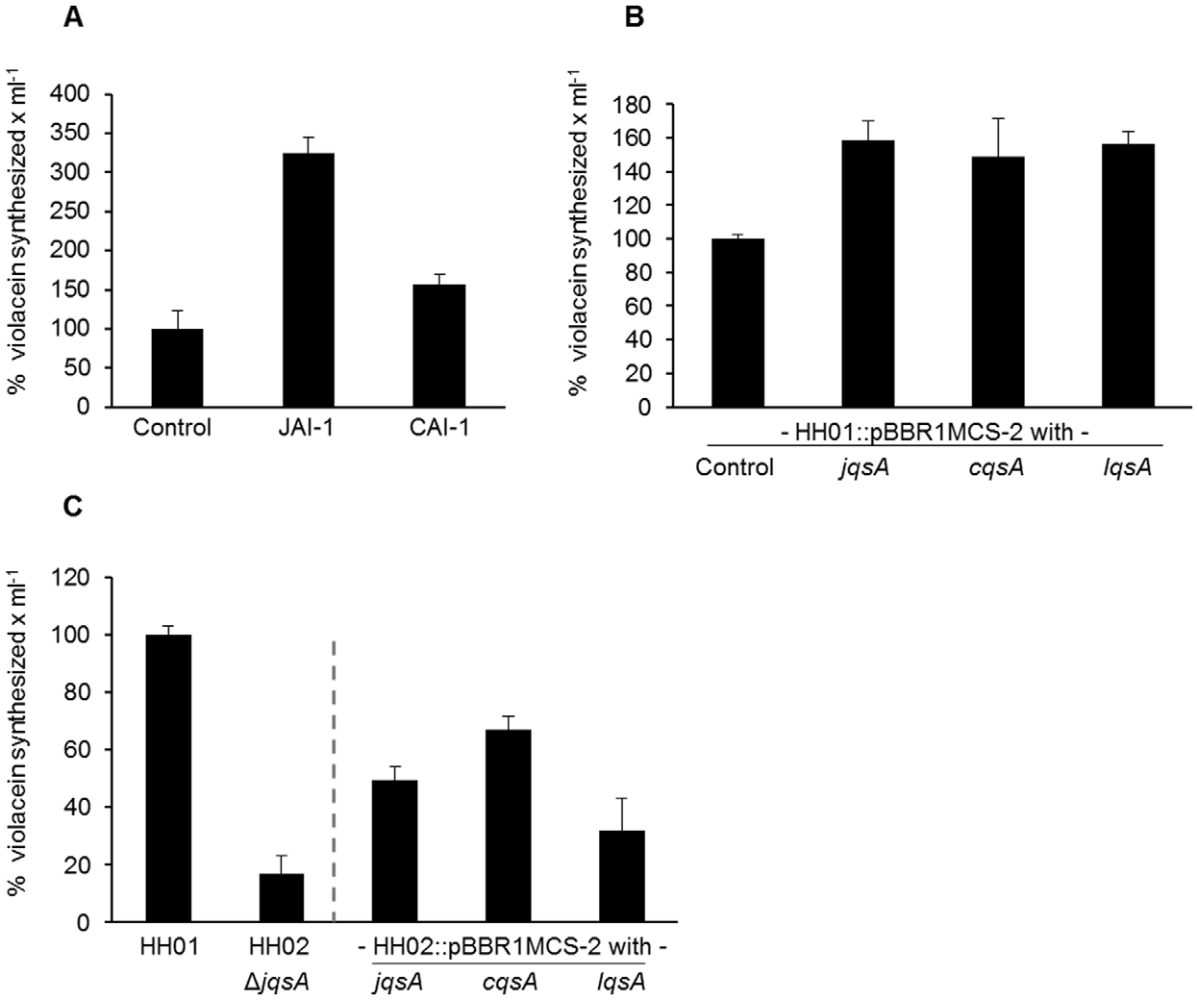
Effect of the known or assumed autoinducer molecules of different strains on the HH01 or HH02 violacein production. A) Effects of extracted possible JAI-1 and CAI-1 autoinducer molecules on HH01 parent strain violacein production. The autoinducers were extracted from *E. coli* DH5α, carrying either the *jqsA* or the *cqsA* gene in pBBR1MCS-2. Autoinducers were purified as described in the material and methods section. 10 µl of these extracts were applied to HH01 growing cultures during early exponential growth phase. The control strain carried the empty vector. B) Effects of extra copies of the HH01 *jqsA, the V. cholerae cqsA* and the *L. pneumophila lqsA* genes in the parent strain HH01. The corresponding genes were inserted into the broad host range vector pBBR1MCS-2 (Table S1). C) Violacein produced by the Δ*jqsA* mutant HH02, HH01 and HH02 carrying either the native *jqsA*, the *V. cholerae cqsA* or the *L. pneumophila lqsA* in pBBR1MCS-2. HH02 carrying an empty pBBR1MCS-2 produced similarly low amounts of violacein compared to HH02 without the empty control vector. Error bars indicate the simple standard deviations. Violacein values were calculated per ml of culture supernatant and normalized with respect to culture density at OD600 nm.

Because of the obvious similarity of JqsA with the *V. cholerae* CqsA and the *L. pneumophila* LqsA we speculated that JqsA is possibly involved in the synthesis of a novel autoinducer molecule, which we designated JAI-1. Furthermore we postulated that this novel autoinducer might be involved in the regulation of the violacein biosynthesis. To verify these hypotheses, we added extracts of *E. coli* cell supernatants overproducing the *jqsA* gene to growing cultures of HH01 and measured the violacein production over time. These tests resulted in a more than 3-fold increased violacein production after 48 h (Fig. 8A). Similarly, extracts of cell supernatants from *E. coli* cells expressing the *V. cholerae cqsA* gene resulted in a 50% increased violacein production in HH01 (Fig. 8A). No such increase was observed, when extracts of supernatants of *E. coli* cells were added that carried the empty vector as control (Fig. 8A). Furthermore, HH01 carrying extra copies of the *jqsA, the cqsA* or the *lqsA* genes produced statistically significant (20–50%) higher amounts of violacein compared to the parent strain (Fig. 8B). In summary, these data supported the notion that the *jqsA* gene is most likely involved in the synthesis of a novel autoinducer molecule. These data also suggested that structurally not yet defined autoinducer molecule affected the violacein biosynthesis in HH01.

To further verify the role of the *jqsA* gene for violacein biosynthesis, we constructed a *jqsA* deletion strain. The corresponding mutant was designated HH02 (Table S1) and it was strongly affected in its capabilities to synthesize violacein. It synthesized 83% less violacein compared to the parent strain (Fig. 8C). As expected, extra copies of the *L. pneumophila lqsA* and the *V. cholerae cqsA* gene partially restored the phenotype of HH02 (Fig. 8C), suggesting that these genes are functional homologues.

Although, we have not yet identified the exact structure of the proposed JAI-1 molecule, we assume that it differs from the published structure for *V. cholerae* and *V. harveyi*. This hypothesis was based on data obtained during complementation tests of *V. cholerae* and *V. harveyi cqsA* mutants. It is notable, that the HH01 derived *jqsA* gene did not complement a *V. cholerae* O1 El Tor *cqsA* mutant (A1552ΔcqsA) with respect to the restoration of at least three tested phenotypes: biofilm formation (lack of repression in Δ*cqsA*), hemagglutinin/protease (Hap) activity and natural transformation (the latter two phenotypes are positively regulated by CAI-1 in the tested *V. cholerae* strain; [21], [97] (Fig. S2). Similar, *V. harveyi* mutants that were deficient in the *cqsA* gene could not be restored with the *jqsA* gene (data not shown). The failure of the JqsA synthesized autoinducer to complement the *V. cholerae* and *V. harveyi* phenotypes might be a result of the relatively high specificity and selectivity of the *cqsA/cqsS* system [99] and these data are in line with earlier observation reported for the C_15_LAI-1 signaling molecule [18]. It is however, noteworthy, that the *L. pneumophila lqsA* expression in *V. cholerae* MM920 triggered the induction of luciferase and thereby suggesting that the signal molecule produced by LqsA is recognized by the corresponding CqsS sensor kinase [18].

Altogether our data suggest that the HH01 *jqsA* gene encodes a homologue of CqsA and LqsA. Although the genome analysis and the initial tests with the *jqsA* deletion strain clearly suggest that *jqsA* is of importance for violacein biosynthesis, the final proof that it synthesizes indeed a novel autoinducer molecule has yet to be furnished. Therefore future work will now have to determine the structure of the postulated JAI-1 signaling molecule and its impact on other regulatory circuits in janthinobacteria and closely related species.

### Conclusions and outlook

The complete genome analysis of HH01 has revealed that its genome shows a high degree of synteny with the already known janthinobacterial genomes. However HH01 also carries several unique features: First, it encodes at least 7 PKS/NRPS clusters. Second, its genome is significantly larger than any of the previously reported janthinobacterial genomes; and third it revealed the presence of an autoinducer system hitherto functionally only characterized in *Vibrio* and *Legionella*. In summary the analysis of the HH01 genome has not only given us a better understanding of the core janthinobacterial genome it has perhaps most importantly, increased the diversity of the known CqsA- and LqsA-like autoinducer synthase proteins. Therefore HH01 might be a suitable model organism for studying the importance of the CqsA/LqsA/JqsA regulatory networks in the background of a non-pathogenic microbe. This appears tempting, since HH01 is genetically accessible and mutations can be generated with relative ease in this microbe. Within this framework it is noteworthy that with the exception of this study only little is known about the ecological role of the CqsA/LqsA/JqsA system in non-pathogenic microbes. Thus future work will now have to unravel the importance of the JqsA/JqsS regulatory circuit with relevance to its importance for survival and growth of this microorganism in its natural aquatic habitat.

## Supporting Information

Figure S1 **Survival of** ***C. elegans*** **in the presence of** ***E. coli*** **DH5α carrying extra copies of the HH01** ***vioA-E*** **genes in pDrive.** For the survival assay 30 L4 larvae were placed onto agar plates. The worms were transferred onto new plates every day and incubated at 20°C. Alive and dead worms were counted during transfer. The treatment groups were violacein expressing *E. coli* DH5α (n = 5) and empty vector *E. coli* DH5α (n = 5) as a control. For experiments with *E.coli* DH5α a single colony was picked, transferred into 100 ml LB medium containing 100 µg/ml ampicillin and incubated on a shaker at 37°C overnight. It was then used to seed NGM Agar plates containing 100 µg/ml ampicillin. 500 µl of the overnight culture was spread onto large plates (φ 9 cm) and 80–90 µl were pipetted into the center of small plates (φ 6 cm). The plates were then incubated at 20°C overnight before use.
(TIF)

Figure S2 ***V. cholerae*** **Δ** ***cqsA*** **mutant cannot be complemented by the HH01** ***jqsA.*** A) The enhanced biofilm formation phenotype of a *V. cholerae* Δ*cqsA* strain cannot be reverted by provision of *jqsA* in trans. The indicated *V. cholerae* strains were incubated statically within 24-well plates and biofilm formation was scored after 24 hours of growth using a standard crystal violet approach. The average of two independent biological replicates with triplicate samples is shown. The error bar indicates the standard deviation. B) The lowered hemagglutinin/protease (Hap) activity of the *ΔcqsA* strain cannot be rescued by *jqsA.* The respective *V. cholerae* strains were grown in LB medium until late exponential phase. At that time aliquots were taken from the culture and the haemagglutinin/protease (Hap) activity was measured using azocasein as a substrate. The average of two independent biological replicates with triplicate samples is shown. C) *JqsA* cannot restore natural transformation in a *V. cholera* Δ*cqsA* mutant. The bacterial strains were tested for chitin-induced natural transformation. Average transformation frequencies of two independent experiments are indicated on the Y-axis. <d.l., below detection limit. *V. cholerae* strains tested in all panels: A1552/pBBR1MCS-2 (WT with vector as control; lanes 1 and 2), ΔcqsA/pBBR1MCS-2 (mutant with vector as control; lanes 3 and 4), Δ*cqsA*/pBBR1MCS2-jqsA (mutant with plasmid containing *jqsA* gene; lanes 5 and 6), and Δ*cqsA*/pBBR1MCS2-cqsA (mutant with plasmid containing *cqsA* gene; lanes 7 and 8). Strains were grown in the absence (odd numbers) or presence (even numbers) of 1 mM IPTG.
(TIF)

Table S1 **Bacterial strains and plasmids used in this study.** amp, ampicillin; gm, gentamycin; nal, nalidxin; km, kanamycin; cyc, cycloserin; tet, tetracycline.
(DOCX)

Table S2 **Primers used for cloning and mutant construction.**
(DOCX)

Table S3 **ORFs and genes predicted in the HH01 genome.** This file contains the submission list of the *Janthinobacterium* sp. HH01 genome. The corresponding GenBank files are available at: DDBJ/EMBL/GenBank access.ion AMWD00000000. Genes/ORFs on contig 1 are indicated with Jab_1cxxxx. Genes/ORFs on contig 2 are indicated with Jab_2cxxxx
(XLSX)

Table S4 **Predicted Genes/ORFs linked to resistance mechanisms in HH01.**
(DOCX)

Table S5 **Predicted genes and ORFs possibly linked to cell appendages and motility in HH01.** Proteins/Genes associated with Type 4 pilus assembly are in blue color.
(DOCX)

Table S6 **Genes/ORFs linked to protein secretion.**
(DOCX)

Table S7 **Secondary metabolite gene clusters in HH01.** NRPS (non-ribosomal peptide synthetases) and PKS (polyketide synthase) proteins are shown in bold. Adenylation (A) with specificity determined by NRPS predictor 2, thiolation (T), condensation (C), condensation/epimerization (C/E), epimerization (E), Coenzyme A ligase (CAL), methyltransferase (MT), thioesterase (TE), reduction (RED), ketosynthase (KS), acyltransferase (AT), ketoreductase (KR).
(DOCX)

Table S8 **HH01 genes possibly linked to cell-cell communication regulatory circuits.**
(DOCX)
