# Supplementary figures and images for: The Janthinobacterium sp. HH01 Genome Encodes a Homologue of the V. cholerae CqsA and L. pneumophila LqsA Autoinducer Synthases
Source: PLoS One. 2013 Feb 6;8(2):e55045. doi: 10.1371/journal.pone.0055045 (PMC3566124; doi:10.1371/journal.pone.0055045)

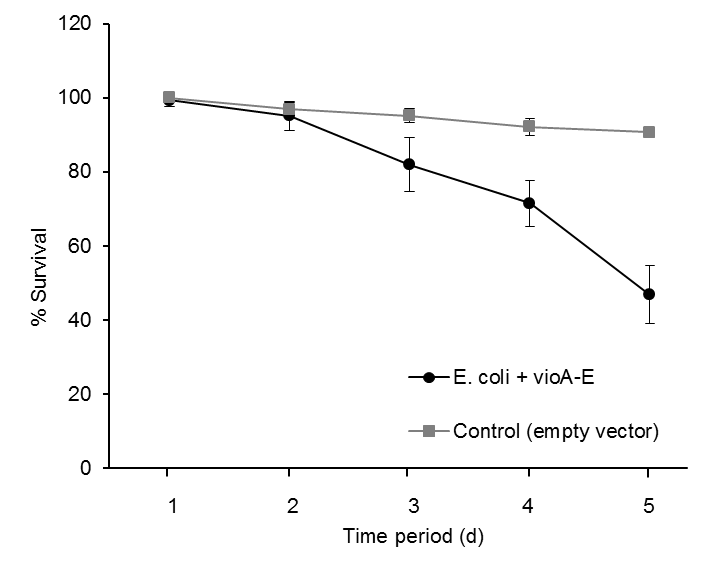

Supplement: Figure S1 — Survival of C. elegans in the presence of E. coli DH5α carrying extra copies of the HH01 vioA-E genes in pDrive. For the survival assay 30 L4 larvae were placed onto agar plates. The worms were transferred onto new plates every day and incubated at 20°C. Alive and dead worms were counted during transfer. The treatment groups were violacein expressing E. coli DH5α (n = 5) and empty vector E. coli DH5α (n = 5) as a control. For experiments with E.coli DH5α a single colony was picked, transferred into 100 ml LB medium containing 100 µg/ml ampicillin and incubated on a shaker at 37°C overnight. It was then used to seed NGM Agar plates containing 100 µg/ml ampicillin. 500 µl of the overnight culture was spread onto large plates (φ 9 cm) and 80–90 µl were pipetted into the center of small plates (φ 6 cm). The plates were then incubated at 20°C overnight before use. (TIF) [file pone.0055045.s001.tif]

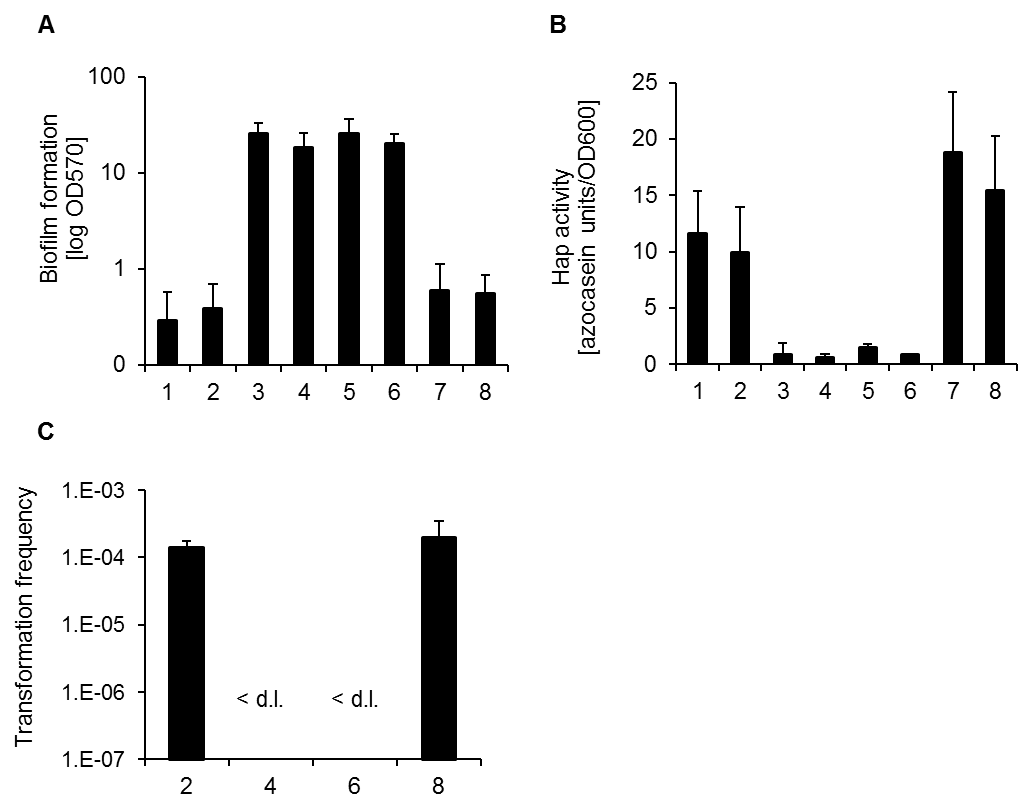

Supplement: Figure S2 — V. cholerae Δ cqsA mutant cannot be complemented by the HH01 jqsA. A) The enhanced biofilm formation phenotype of a V. cholerae ΔcqsA strain cannot be reverted by provision of jqsA in trans. The indicated V. cholerae strains were incubated statically within 24-well plates and biofilm formation was scored after 24 hours of growth using a standard crystal violet approach. The average of two independent biological replicates with triplicate samples is shown. The error bar indicates the standard deviation. B) The lowered hemagglutinin/protease (Hap) activity of the ΔcqsA strain cannot be rescued by jqsA. The respective V. cholerae strains were grown in LB medium until late exponential phase. At that time aliquots were taken from the culture and the haemagglutinin/protease (Hap) activity was measured using azocasein as a substrate. The average of two independent biological replicates with triplicate samples is shown. C) JqsA cannot restore natural transformation in a V. cholera ΔcqsA mutant. The bacterial strains were tested for chitin-induced natural transformation. Average transformation frequencies of two independent experiments are indicated on the Y-axis. <d.l., below detection limit. V. cholerae strains tested in all panels: A1552/pBBR1MCS-2 (WT with vector as control; lanes 1 and 2), ΔcqsA/pBBR1MCS-2 (mutant with vector as control; lanes 3 and 4), ΔcqsA/pBBR1MCS2-jqsA (mutant with plasmid containing jqsA gene; lanes 5 and 6), and ΔcqsA/pBBR1MCS2-cqsA (mutant with plasmid containing cqsA gene; lanes 7 and 8). Strains were grown in the absence (odd numbers) or presence (even numbers) of 1 mM IPTG. (TIF) [file pone.0055045.s002.tif]
