# Supplementary material for: The Janthinobacterium sp. HH01 Genome Encodes a Homologue of the V. cholerae CqsA and L. pneumophila LqsA Autoinducer Synthases
Source: PLoS One. 2013 Feb 6;8(2):e55045. doi: 10.1371/journal.pone.0055045 (PMC3566124; doi:10.1371/journal.pone.0055045)
Supplement: Table S1 — Bacterial strains and plasmids used in this study. amp, ampicillin; gm, gentamycin; nal, nalidxin; km, kanamycin; cyc, cycloserin; tet, tetracycline. (DOCX) [file pone.0055045.s003.docx]

| **Strain or construct** | **Description** | **Reference/Source** |
| --- | --- | --- |
| *Strains* |  |  |
| *Janthinobacterium* sp. HH01 | Wildtype isolate, amp*^R^*, cm*^R^*, tet*^R^*, nal*^R^*, cyc*^R^,gm^s^, km^s^* | This work |
| *Janthinobacterium* sp. HH02 | *ΔjqsA mutant of HH01, gm^R^* | This work |
| *Janthinobacterium* sp. MP5059B | Wildtype strain | H. Bode lab |
| *Janthinobacterium* sp. HH5-1 | EZ-Tn5TM kan^R^, *trpF* of HH01*, trp^-^, vio^-^* | This work |
| *Duganella violaceinigra* DSMZ #15887 | Wildtype strain | DSMZ, Braunschweig, Germany |
| *Vibrio cholerae* A1552 | *Vibrio cholerae* O1 El Tor | [20] |
| *Vibrio cholerae* A1552*ΔcqsA* | A1552*Δ VCA0523 (csqA)* | [21] |
| *Vibrio harveyi* BB120 | Wildtype strain | [22] |
| *Vibrio harveyi* MR14 | *ΔcqsA, ΔluxS mutant of V. harveyi strain BB-120 carrying the HH01 cqsA gene in pBRMCS1-2* | This work |
| *E. coli* OP50 | Derivative of *E. coli*(B) Berkeley strain, *ura*^-^, str^R^ | [23] |
| *E. coli* Epi300 | Host strain for fosmid libraries | Epicentre, Madison, WI, USA |
| *E. coli* DH5α | Cloning strain, *recA1*, Δl*acZ* | Invitrogen, Darmstadt, Germany |
| *E. coli*S17-1 λpir | Modified RP4 plasmid integrated into genome | [24] |
| *Plasmids* |  |  |
| pBBR1MCS-2 | Broad host-range vector, km^R^ | [25] |
| pBBR1MCS-5 | Broad host-range vector, gm^R^ | [25] |
| pBBRCH-jqsA | *jqsA* in pBBR1MCS-2 | This work |
| pBBR-cqsA_VH | pBBR1MCS-2 with *V. harveyi* BB120 *cqsA* under control of the arabinose promoter | This work |
| pTS21 | Legionella sp*. lqsA* in pET28 | [26] |
| pBBRCH-lqsA | *lqsA* in pBBR1MCS2-in *XhoI-BamHI* site | This work |
| pBBRCH-cqsA | *cqsA* from *V. cholerae* A1552 integrated into plasmid pBBR1MCS-2 | This work |
| pDrive | PCR cloning vector, amp^R^, km^R^ | Qiagen, Hilden, Germany |
| pDrive-vioABCDE | pDrive encoding the *vioA-E* genes under the native promoter | This work |
| pNPTS138-R6KT | Suicide vector for knock outs | [27] |
