# Supplementary material for: The Janthinobacterium sp. HH01 Genome Encodes a Homologue of the V. cholerae CqsA and L. pneumophila LqsA Autoinducer Synthases
Source: PLoS One. 2013 Feb 6;8(2):e55045. doi: 10.1371/journal.pone.0055045 (PMC3566124; doi:10.1371/journal.pone.0055045)
Supplement: Table S2 — Primers used for cloning and mutant construction. (DOCX) [file pone.0055045.s004.docx]

| **Primer** | **Sequence (5´-3´)** | **Reference** |
| --- | --- | --- |
| *JqsA*cloningprimer_for | TTGCCATGATCGCCTTAAAT | This work |
| *JqsA*cloningprimer_rev | ACCTCCATGATAGCGTACCG | This work |
| *jqsA* deletionmutant_A1_*Bam*HI | CTTAGGATCCACCTCCATGATAGCGTACCG | This work |
| *jqsA*deletion mutant_A2_*Xba*I | CGATTCTAGATCAAGGGCTACTTCCTGTCC | This work |
| *jqsA*deletion mutant_B1_*Xba*I | GCTATCTAGATGTCCAGGTAGACCGGAATG | This work |
| j*qsA*deletion mutant_B2_*Eco*RI | GTCCGAATTCAAGTGCTGCCAGATCACGTA | This work |
| Gm^R^_for_*Xba*I | GACATCTAGAGACGCACACCGTGGAAAC | This work |
| Gm^R^_rev_*Xba*I | TAATCTAGACCGCGATCATCAAGGCCGTG | This work |
| j*qsA*mutant_control_outA | GTCTCGACGTTCTTCGCATACT | This work |
| j*qsA*mutant_control_outB | GTGGTGTCGTGGAAGGTCTG | This work |
| j*qsA* mutant_control_rev1 | TGTCCAGGTAGACCGGAATG | This work |
| j*qsA* mutant_control_rev2 | GCACAACGACATCGAACATC | This work |
| Inv-2 | GAACTTTTGCTGAGTTGAAGGATCA | [28] |
| KAN-2 FP-1 | ACCTACAACAAAGCTCTCATCAACC | Epicentre, Madison, Wisconsin, USA |
