# Supplementary material for: The Janthinobacterium sp. HH01 Genome Encodes a Homologue of the V. cholerae CqsA and L. pneumophila LqsA Autoinducer Synthases
Source: PLoS One. 2013 Feb 6;8(2):e55045. doi: 10.1371/journal.pone.0055045 (PMC3566124; doi:10.1371/journal.pone.0055045)
Supplement: Table S4 — Predicted Genes/ORFs linked to resistance mechanisms in HH01. (DOCX) [file pone.0055045.s006.docx]

| **Resistance** | **ORF/Locus** | **Predicted function** |
| --- | --- | --- |
| Tetracycline |  |  |
|  | Jab_1c02970 | tetracycline resistance protein class A |
|  | Jab_1c09990 | tetracycline resistance protein class A |
|  | Jab_2c01640 | tetracycline resistance protein class A |
| Chloramphenicol |  |  |
|  | Jab_2c08860 | chloramphenicol resistance protein CmlR |
| Virginiamycin A / Streptogramin A |  |  |
|  | Jab_2c00560 | virginiamycin A acetyltransferase |
| Fusaric acid |  |  |
|  | Jab_1c18420 | fusaric acid resistance family protein |
|  | Jab_1c18440 | fusaric acid resistance protein FusA |
| Fosmidomycin |  |  |
|  | Jab_2c15720 | fosmidomycin resistance protein Fsr |
|  | Jab_2c15980 | fosmidomycin resistance protein Fsr |
| Methylenomycin A |  |  |
|  | Jab_2c16850 | methylenomycin A resistance protein Mmr |
| Bicyclomycin resistance |  |  |
|  | Jab_2c13540 | bicyclomycin resistance protein |
| Glyoxalase/bleomycin resistance protein/dioxygenase |  |  |
|  | Jab_1c04360 | glyoxalase/bleomycin resistance |
|  | Jab_1c22410 | glyoxalase/bleomycin resistance protein/dioxygenase |
|  | Jab_1c24250 | glyoxalase/bleomycin resistance  protein/dioxygenase |
|  | Jab_2c05770 | glyoxalase/bleomycin resistance protein/dioxygenase |
|  | Jab_2c09850 | glyoxalase/bleomycin resistance protein/dioxygenase |
|  | Jab_2c16240 | glyoxalase/bleomycin resistance protein/dioxygenase |
|  | Jab_2c16800 | glyoxalase/bleomycin resistance protein/dioxygenase |
|  | Jab_2c18920 | glyoxalase/bleomycin resistance protein/dioxygenase |
|  | Jab_2c22090 | glyoxalase/bleomycin resistance protein/dioxygenase |
|  | Jab_2c22530 | glyoxalase/bleomycin resistance protein/dioxygenase |
|  | Jab_2c22580 | glyoxalase/bleomycin resistance protein/dioxygenase |
|  | Jab_2c22590 | glyoxalase/bleomycin resistance protein/dioxygenase |
|  | Jab_2c23310 | glyoxalase/bleomycin resistance protein/dioxygenase |
|  | Jab_2c32610 | glyoxalase/bleomycin resistance protein/dioxygenase |
|  | Jab_2c35200 | glyoxalase/bleomycin resistance protein/dioxygenase |
| Acriflavin/multi resistance |  |  |
|  | Jab_1c04940 | major facilitator superfamily MFS_1 transporter |
|  | Jab_1c07040 | RND family efflux transporter MFP subunit |
|  | Jab_1c07560 | multidrug resistance protein MdtA |
|  | Jab_1c07570 | multidrug resistance protein MdtC |
|  | Jab_1c10490 | drug resistance transporter EmrB/QacA subfamily |
|  | Jab_1c16460 | major facilitator superfamily MFS_1 transporter |
|  | Jab_1c16610 | drug resistance transporter EmrB/QacA subfamily |
|  | Jab_1c17160 | major facilitator superfamily MFS_1 protein |
|  | Jab_1c17500 | multidrug resistance protein PmpM |
|  | Jab_1c17920 | MotA/TolQ/ExbB proton channel family protein |
|  | Jab_1c17930 | biopolymer transport protein ExbD/TolR |
|  | Jab_1c17940 | multidrug resistance protein MdtA |
|  | Jab_1c17950 | multidrug resistance protein MdtC |
|  | Jab_1c20500 | efflux transporter, RND family, MFP subunit |
|  | Jab_1c20510 | acriflavin resistance protein |
|  | Jab_1c21530 | multidrug resistance protein MdtB |
|  | Jab_1c21540 | AcrB/AcrD/AcrF family protein |
|  | Jab_1c23970 | major facilitator superfamily MFS_1 protein |
|  | Jab_1c24060 | major facilitator superfamily MFS_1 protein |
|  | Jab_2c00300 | multidrug resistance protein stp |
|  | Jab_2c00960 | acriflavine resistance protein A |
|  | Jab_2c00970 | efflux pump membrane transporter BepE |
|  | Jab_2c00980 | outer membrane lipoprotein efflux system NodT family |
|  | Jab_2c04530 | ABC-type multidrug transport system, ATPase and permease |
|  | Jab_2c05190 | multidrug resistance protein stp |
|  | Jab_2c05830 | transmembrane protein drug efflux protein |
|  | Jab_2c06180 | MFS-type transporter |
|  | Jab_2c07220 | major facilitator superfamily MFS_1 |
|  | Jab_2c09110 | major facilitator superfamily MFS_1 |
|  | Jab_2c10480 | acriflavine resistance protein E |
|  | Jab_2c10490 | acriflavine resistance protein F |
|  | Jab_2c10500 | acriflavine resistance protein F |
|  | Jab_2c10510 | RND efflux system outer membrane lipoprotein |
|  | Jab_2c14680 | putative multidrug efflux protein |
|  | Jab_2c14700 | putative efflux pump outer membrane protein |
|  | Jab_2c17410 | RND efflux system, outer membrane lipoprotein NodT |
|  | Jab_2c17420 | acriflavin resistance protein MdtC |
|  | Jab_2c17430 | acriflavin resistance protein MdtB |
|  | Jab_2c17440 | acriflavin resistance protein MdtA |
|  | Jab_2c17510 | major facilitator superfamily MFS_1 |
|  | Jab_2c18450 | antibiotic biosynthesis monooxygenase |
|  | Jab_2c18830 | efflux transporter RND family |
|  | Jab_2c18840 | macrolide ABC transport system ATP-binding protein |
|  | Jab_2c18850 | macrolide ABC transport system permease protein |
|  | Jab_2c18860 | macrolide ABC transport system permease protein |
|  | Jab_2c19420 | multiple antibiotic resistance MarC-like protein |
|  | Jab_2c20210 | multidrug ABC transport system ATP-binding protein |
|  | Jab_2c20390 | small multidrug resistance protein |
|  | Jab_2c20400 | small multidrug resistance protein |
|  | Jab_2c20520 | RND efflux system outer membrane lipoprotein NodT family |
|  | Jab_2c20530 | acriflavine resistance protein B |
|  | Jab_2c20540 | acriflavine resistance protein A |
|  | Jab_2c21150 | small multidrug resistance protein |
|  | Jab_2c21510 | major facilitator superfamily MFS_1 |
|  | Jab_2c23460 | acriflavine resistance protein A |
|  | Jab_2c23470 | acriflavine resistance protein B |
|  | Jab_2c23480 | antibiotic efflux pump outer membrane protein ArpC |
|  | Jab_2c23590 | antibiotic biosynthesis monooxygenase |
|  | Jab_2c24280 | major facilitator superfamily MFS_1 |
|  | Jab_2c25080 | major facilitator superfamily MFS_1 |
|  | Jab_2c25190 | major facilitator superfamily MFS_1 |
|  | Jab_2c25270 | multidrug resistance protein MdtC |
|  | Jab_2c25280 | multidrug resistance protein A |
|  | Jab_2c26060 | multidrug ABC transport system ATP-binding protein mdlA |
|  | Jab_2c26070 | multidrug ABC transport system ATP-binding protein mdlB |
|  | Jab_2c26250 | RND efflux system NodT family |
|  | Jab_2c25260 | RND efflux system |
|  | Jab_2c26270 | multidrug resistance protein MdtC |
|  | Jab_2c26310 | major facilitator superfamily MFS_1 |
|  | Jab_2c26290 | efflux transporter outer membrane factor lipoprotein, NodT family |
|  | Jab_2c27780 | multidrug resistance protein MdtC |
|  | Jab_2c27770 | RND efflux pump membrane fusion protei |
|  | Jab_2c28140 | major facilitator superfamily MFS_1 |
|  | Jab_2c29980 | major facilitator superfamily MFS_1 |
|  | Jab_2c30500 | efflux transporter, RND family, MFP subunit |
|  | Jab_2c31420 | major facilitator superfamily MFS_1 |
|  | Jab_2c32510 | drug resistance transporter Bcr/CflA subfamily |
|  | Jab_2c33560 | major facilitator superfamily MFS_1 transporter |
|  | Jab_2c35340 | MATE efflux family protein |
| Ampicilin/Pennicillin |  |  |
|  | Jab_1c09640 | predicted beta-lactamase |
|  | Jab_1c21610 | predicted beta-lactamase |
|  | Jab_2c07130 | predicted beta-lactamase |
|  | Jab_2c07700 | predicted beta-lactamase |
|  | Jab_2c14380 | predicted beta-lactamase |
|  | Jab_2c15870 | predicted beta-lactamase |
|  | Jab_2c15930 | predicted beta-lactamase |
|  | Jab_2c21590 | predicted beta-lactamase |
|  | Jab_2c23440 | predicted beta-lactamase |
| Cobalt/zinc/cadmium/copper |  |  |
|  | Jab_2c30760 | cobalt-zinc-cadmium efflux system protein CzcD |
|  | Jab_2c35240 | cation efflux system protein CzcA |
|  | Jab_2c35250 | cobalt-zinc-cadmium resistance protein CzcB |
|  | **Jab_2c03840 -** | ***Gene cluster linked to heavy metal detoxification*** |
|  | **Jab_2c04220** | ***38 genes/ORFs in total*** |
|  | Jab_2c06820 | putative multicopper oxidase |
|  | Jab_2c06830 | copper resistance protein CopC |
|  | Jab_2c15040 | cobalt-zinc-cadmium resistance protein czcB |
|  | Jab_2c15050 | cation efflux system protein czcA |
| Telluride |  |  |
|  | Jab_1c13010 | tellurium resistance protein TerE |
|  | Jab_1c13030 | tellurium resistance protein TerZ |
| Chromate |  |  |
|  | Jab_2c15180 | chromate transporter |
|  | Jab_1c19570 | chromate transporter |
|  | Jab_2c33450 | chromate transporter |
|  | Jab_2c33460 | chromate transporter |
|  |  |  |
| Arsenate/arsenic acid |  |  |
|  | Jab_1c01270 | arsenical resistance protein ArsH |
|  | Jab_1c01280 | arsenical pump membrane protein ArsB |
|  | Jab_1c01290 | arsenate reductase ArsC |
|  | Jab_1c14880 | arsenate reductase ArsC |
| Ammonium |  |  |
|  | Jab_2c01340 | quaternary ammonium compound-resistance protein SugE |
