# Supplementary material for: The Janthinobacterium sp. HH01 Genome Encodes a Homologue of the V. cholerae CqsA and L. pneumophila LqsA Autoinducer Synthases
Source: PLoS One. 2013 Feb 6;8(2):e55045. doi: 10.1371/journal.pone.0055045 (PMC3566124; doi:10.1371/journal.pone.0055045)
Supplement: Table S5 — Predicted genes and ORFs possibly linked to cell appendages and motility in HH01. Proteins/Genes associated with Type 4 pilus assembly are in blue color. (DOCX) [file pone.0055045.s007.docx]

| **Locus tag** | **Gene** | **Predicted function** |
| --- | --- | --- |
| Jab_1c02000 |  | methyl-accepting chemotaxis sensory transducer with PAS/PAC sensor |
| Jab_1c02650 |  | methyl-accepting chemotaxis sensory transducer |
| Jab_1c04480 |  | methyl-accepting chemotaxis protein |
| Jab_1c04690 | *cheB1* | chemotaxis protein-glutamate methylesterase CheB |
| Jab_1c05080 |  | Flp/Fap pilin component family protein |
| Jab_1c05120 | *cpaB* | Flp pilus assembly protein CpaB |
| Jab_1c06200 |  | methyl-accepting chemotaxis protein |
| Jab_1c06210 | *cheW1* | chemotaxis protein CheW |
| Jab_1c06530 |  | methyl-accepting chemotaxis protein |
| Jab_1c06560 |  | methyl-accepting chemotaxis protein |
| Jab_1c06570 | *cheY5* | putative chemotaxis protein CheY |
| Jab_1c06590 | *cheA1* | chemotaxis protein CheA |
| Jab_1c06600 | *cheW2* | chemotaxis protein CheW |
| Jab_1c06610 | *cheR1* | chemotaxis protein methyltransferase CheR |
| Jab_1c06620 | *cheD* | chemoreceptor glutamine deamidase CheD |
| Jab_1c06630 | *cheB2* | chemotaxis response regulator methylesterase CheB |
| Jab_1c06650 |  | methyl-accepting chemotaxis protein |
| Jab_1c06660 | *cheW3* | CheW protein |
| Jab_1c06670 |  | methyl-accepting chemotaxis protein |
| Jab_1c06680 |  | putative methyl-accepting chemotaxis protein |
| Jab_1c07780 | *pilM* | type IV pilus assembly protein PilM |
| Jab_1c07790 | *pilN* | type IV pilus assembly protein PilN |
| Jab_1c07800 | *pilO* | type IV pilus assembly protein PilO |
| Jab_1c07810 | *pilP* | type IV pilus assembly protein PilP |
| Jab_1c07820 | *pilQ* | type IV pilus assembly protein PilQ |
| Jab_1c08760 |  | methyl-accepting chemotaxis sensory transducer |
| Jab_1c08800 |  | P pilus assembly protein chaperone PapD-like protein |
| Jab_1c08810 |  | fimbrial biogenesis outer membrane usher protein |
| Jab_1c09390 |  | methyl-accepting chemotaxis sensory transducer |
| Jab_1c10060 | *rssB* | swarming motility regulation protein RssB |
| Jab_1c11770 | *tsr1* | methyl-accepting chemotaxis protein I |
| Jab_1c1229 | *flhA* | S-(hydroxymethyl)glutathione dehydrogenase FlhA |
| Jab_1c12550 | *pilT1* | twitching mobility protein PilT |
| Jab_1c12560 | *pilT2* | twitching mobility protein PilT |
| Jab_1c12670 | *pilT3* | twitching mobility protein PilT |
| Jab_1c13820 | *pilD* | type IV prepilin-like proteins leader peptide-processing protein PilD |
| Jab_1c13860 | *pilC1* | type IV fimbrial assembly protein PilC1 |
| Jab_1c13870 | *pilB* | type IV fimbrial assembly protein PilB |
| Jab_1c14040 |  | putative methyl-accepting chemotaxis receptor |
| Jab_1c15550 | *tsr2* | methyl-accepting chemotaxis protein I |
| Jab_1c17430 | *fliT* | FliT flagellar protein |
| Jab_1c20230 | *cheB3* | chemotaxis response regulator methylesterase CheB |
| Jab_1c20240 | *cheA2* | signal transduction histidine kinase cheA |
| Jab_1c20250 | *cheW4* | chemotaxis protein CheW |
| Jab_1c20260 | *cheR2* | chemotaxis protein methyltransferase CheR |
| Jab_1c20270 | *cheW5* | chemotaxis protein CheW |
| Jab_1c20280 |  | methyl-accepting chemotaxis sensory transducer |
| Jab_1c20570 |  | methyl-accepting chemotaxis protein |
| Jab_1c20670 |  | methyl-accepting chemotaxis protein |
| Jab_1c21010 | *pilV* | type IV pilus assembly protein PilV |
| Jab_1c21020 | *pilW1* | type IV pilus assembly protein PilW1 |
| Jab_1c21030 | *pilX* | type IV pilus assembly protein PilX |
| Jab_1c21040 | *pilY* | type IV pilus assembly protein, Tip-like adhesin PilY |
| Jab_1c21050 | *pilE* | type IV pilus assembly protein PilE |
| Jab_1c21060 | *fimT* | type IV pilus assembly protein FimT |
| Jab_1c23710 |  | methyl-accepting chemotaxis protein |
| Jab_1c23890 |  | methyl-accepting chemotaxis protein |
| Jab_1c24400 |  | flagellar hook 2 domain-containing protein |
| Jab_2c00570 | *tse1* | methyl-accepting chemotaxis serine transducer |
| Jab_2c01670 | *cheW6* | chemotaxis protein CheW |
| Jab_2c01680 | *tar1* | methyl-accepting chemotaxis protein II |
| Jab_2c02410 | *tsr1* | methyl-accepting chemotaxis protein I |
| Jab_2c02580 | *pilC2* | type IV pilus assembly protein PilC2 |
| Jab_2c02650 | *pilC3* | type IV fimbrial assembly protein PilC3 |
| Jab_2c03250 | *tsr2* | methyl-accepting chemotaxis protein I |
| Jab_2c04070 | *flgB1* | flagellar basal-body rod protein FlgB |
| Jab_2c06210 | *cheW2* | chemotaxis protein CheW |
| Jab_2c06230 | *cheR3* | chemotaxis protein methyltransferase 1 |
| Jab_2c06240 | *mcpA* | methyl-accepting chemotaxis protein McpA |
| Jab_2c06450 | *pilZ* | type IV pilus assembly protein PilZ |
| Jab_2c06640 | *rssB* | swarming motility regulation protein RssB |
| Jab_2c06650 | *rssA* | swarming motility regulation sensor protein RssA |
| Jab_2c08550 | *tsr3* | methyl-accepting chemotaxis protein I |
| Jab_2c08730 | *tsr4* | methyl-accepting chemotaxis protein I |
| Jab_2c08760 | *cheW7* | chemotaxis protein CheW |
| Jab_2c09540 | *tar2* | methyl-accepting chemotaxis protein II |
| Jab_2c12000 | *tsr5* | methyl-accepting chemotaxis protein I |
| Jab_2c12090 | *tar3* | methyl-accepting chemotaxis protein II |
| Jab_2c12210 | *pilW3* | type IV pilus assembly protein pilW3 |
| Jab_2c12240 |  | putative fimbrial protein |
| Jab_2c12550 |  | flagellin domain protein |
| Jab_2c12580 | *fhiA* | putative protein FhiA |
| Jab_2c12590 | *flhB1* | flagellar biosynthetic protein FlhB |
| Jab_2c12600 | *fliR1* | flagellar biosynthetic protein FliR |
| Jab_2c12610 | *fliQ1* | flagellar biosynthetic protein FliQ |
| Jab_2c12620 | *fliP1* | flagellar biosynthetic protein FliP |
| Jab_2c12630 | *fliN1* | flagellar motor switch protein FliN |
| Jab_2c12660 | *fliF1* | flagellar M-ring protein FliF |
| Jab_2c12670 | *fliG1* | flagellar motor switch protein FliG |
| Jab_2c12680 |  | putative flagellar assembly protein |
| Jab_2c12690 | *fliI1* | flagellum-specific ATP synthase FliI |
| Jab_2c12710 | *fliDL* | lateral flagellar hook protein 2 |
| Jab_2c12720 | *fliS* | B-type flagellar protein fliS, putative |
| Jab_2c12740 |  | flagellar hook-length control protein |
| Jab_2c12750 |  | flagellar basal body protein |
| Jab_2c12760 | *lafS* | RNA polymerase sigma factor for flagellar operon |
| Jab_2c12770 | *lafT* | chemotaxis protein LafT |
| Jab_2c12780 | *lafU* | chemotaxis protein LafU |
| Jab_2c12810 | *flgJ1* | peptidoglycan hydrolase flgJ |
| Jab_2c12820 | *flgA1* | flagella basal body P-ring formation protein FlgA |
| Jab_2c12830 | *flgB2* | flagellar basal-body rod protein FlgB |
| Jab_2c12840 | *flgC1* | flagellar basal-body rod protein FlgC |
| Jab_2c12850 | *flgD* | basal-body rod modification protein FlgD, putative |
| Jab_2c12860 | *flgE1* | flagellar hook protein FlgE |
| Jab_2c12870 | *flgF1* | flagellar basal-body rod protein FlgF |
| Jab_2c12880 | *flgG1* | flagellar basal-body rod protein FlgG |
| Jab_2c12890 | *flgH1* | flagellar L-ring protein FlgH |
| Jab_2c12900 | *flgI1* | flagellar P-ring protein FlgI |
| Jab_2c12920 | *flgK1* | flagellar hook protein FlgK |
| Jab_2c12930 | *flgL1* | flagellar hook protein FlgL |
| Jab_2c12970 | *fliY* | cystine-binding periplasmic protein FliY |
| Jab_2c13240 |  | chemotaxis protein methyltransferase |
| Jab_2c13940 | *cheV* | chemotaxis protein CheV |
| Jab_2c14830 | *tar4* | methyl-accepting chemotaxis protein II |
| Jab_2c15150 | *tsr6* | methyl-accepting chemotaxis protein I |
| Jab_2c16460 | *cheZ1* | protein phosphatase CheZ |
| Jab_2c16470 | *cheY1* | chemotaxis protein CheY |
| Jab_2c16480 | *cheB* | chemotaxis response regulator protein-glutamate methylesterase CheB |
| Jab_2c16490 | *cheD* | chemoreceptor glutamine deamidase CheD |
| Jab_2c16500 | *cheR4* | chemotaxis protein methyltransferase CheR |
| Jab_2c16510 | *tsr7* | methyl-accepting chemotaxis protein I |
| Jab_2c16520 | *cheW8* | chemotaxis protein CheW |
| Jab_2c16530 | *cheA1* | chemotaxis protein CheA |
| Jab_2c16540 | *cheY2* | chemotactic response regulator CheY |
| Jab_2c16560 | *motB1* | flagellar motor protein MotB |
| Jab_2c16570 | *motA1* | flagellar motor protein MotA |
| Jab_2c16590 | *flhC* | flagellar transcriptional activator FlhC |
| Jab_2c16600 | *flhD* | transcriptional activator FlhD |
| Jab_2c17100 | *pilT4* | twitching mobility protein PilT4 |
| Jab_2c17190 | *cheW9* | chemotaxis protein CheW |
| Jab_2c18270 | *tsr8* | methyl-accepting chemotaxis protein I |
| Jab_2c19020 | *tsr9* | methyl-accepting chemotaxis protein I |
| Jab_2c19230 | *tsr10* | methyl-accepting chemotaxis protein I |
| Jab_2c19590 | *pilW2* | type IV pilus biogenesis/stability protein PilW2 |
| Jab_2c20990 | *tsr11* | methyl-accepting chemotaxis protein I |
| Jab_2c21060 | *tar5* | methyl-accepting chemotaxis protein II |
| Jab_2c21730 | *fimV* | type IV pilus assembly protein FimV |
| Jab_2c21790 | *cheA2* | signal transduction histidine kinase CheA |
| Jab_2c21800 | *cheZ2* | protein phosphatase CheZ |
| Jab_2c23280 | *cheC* | CheY-P phosphatase CheC |
| Jab_2c23500 | *tsr12* | methyl-accepting chemotaxis protein I |
| Jab_2c23710 | *fliC* | flagellin FliC |
| Jab_2c23720 | *flaG* | flagellar protein FlaG |
| Jab_2c23730 | *fliD* | flagellar hook protein 2 |
| Jab_2c23740 | *fliS* | flagellar protein FliS |
| Jab_2c23750 | *fliT* | flagellar protein FliT |
| Jab_2c23760 | *fliK1* | flagellar hook-length control protein FliK |
| Jab_2c23770 | *flhB2* | flagellar biosynthetic protein FlhB |
| Jab_2c23780 | *ycgR* | flagellar brake protein YcgR |
| Jab_2c23790 | *fliE* | flagellar hook-basal body complex subunit FliE |
| Jab_2c23800 | *fliF2* | flagellar M-ring protein FliF |
| Jab_2c23810 | *fliG2* | flagellar motor switch protein FliG |
| Jab_2c23820 | *fliH* | flagellar assembly protein FliH |
| Jab_2c23830 | *fliI2* | flagellum-specific ATP synthase FliI |
| Jab_2c23840 | *fliJ* | flagellar export protein FliJ |
| Jab_2c23850 | *fliK2* | flagellar hook-length control protein FliK |
| Jab_2c23860 | *fliL* | flagellar basal body protein FliL |
| Jab_2c23870 | *fliM* | flagellar motor switch protein FliM |
| Jab_2c23880 | *fliN2* | flagellar motor switch protein FliN |
| Jab_2c23890 | *fliO* | flagellar biosynthesis protein FliO |
| Jab_2c23900 | *fliP2* | flagellar biosynthesis protein FliP |
| Jab_2c23910 | *fliQ2* | flagellar biosynthesis protein FliQ |
| Jab_2c23920 | *fliR2* | flagellar biosynthesis protein FliR |
| Jab_2c23930 | *frpC* | iron-regulated protein FrpC |
| Jab_2c23940 | *flgL2* | flagellar hook protein FlgL |
| Jab_2c23950 | *flgK2* | flagellar hook protein FlgK |
| Jab_2c23960 | *flgJ2* | flagellar rod assembly protein/muramidase FlgJ |
| Jab_2c23970 | *flgI2* | flagellar P-ring protein FlgI |
| Jab_2c23980 | *flgH2* | flagellar L-ring protein FlgH |
| Jab_2c23990 | *flgG2* | flagellar basal-body rod protein FlgG |
| Jab_2c24000 | *flgF2* | flagellar basal-body rod protein FlgF |
| Jab_2c24010 | *flgE2* | flagellar hook protein FlgE |
| Jab_2c24020 | *flgD* | flagellar hook capping protein FlgD |
| Jab_2c24030 | *flgC2* | flagellar basal-body rod protein FlgC |
| Jab_2c24040 | *flgB3* | flagellar basal-body rod protein FlgB |
| Jab_2c24050 | *flgA2* | flagella basal body P-ring formation protein FlgA |
| Jab_2c24060 | *flgM* | flagellar biosynthesis anti-sigma factor FlgM |
| Jab_2c24070 | *flgN* | flagellar synthesis protein FlgN |
| Jab_2c24080 | *motB2* | flagellar motor protein MotB |
| Jab_2c24090 | *motA2* | flagellar motor protein MotA |
| Jab_2c24100 | *fliA* | flagellar biosynthesis sigma factor FliA |
| Jab_2c24110 | *fleN* | flagellar synthesis regulator FleN |
| Jab_2c24120 | *flhF* | flagellar biosynthesis regulator FlhF |
| Jab_2c24130 |  | transposase |
| Jab_2c24140 | *flhA* | flagellar biosynthesis protein FlhA |
| Jab_2c24150 | *flhB3* | flagellar biosynthesis protein FlhB |
| Jab_2c24680 | *tsr13* | methyl-accepting chemotaxis protein I |
| Jab_2c26000 | *tsr14* | methyl-accepting chemotaxis protein I |
| Jab_2c27030 | *tsr15* | methyl-accepting chemotaxis protein I |
| Jab_2c27420 | *tsr16* | methyl-accepting chemotaxis protein I |
| Jab_2c28650 | *cheA3* | chemotaxis protein CheA |
| Jab_2c28660 | *pilJ* | methyl-accepting chemotaxis protein I |
| Jab_2c28670 | *cheW6* | chemotaxis protein CheW |
| Jab_2c28680 | *cheY3* | signal transduction response regulator |
| Jab_2c28690 | *cheY4* | signal transduction response regulator |
| Jab_2c30010 | *tar6* | methyl-accepting chemotaxis protein II |
| Jab_2c32460 | *fla* | flagellin |
| Jab_2c34790 | *tse2* | methyl-accepting chemotaxis serine transducer |
| Jab_2c34810 | *tsr17* | methyl-accepting chemotaxis protein I |
| Jab_1c04790 | *aer* | aerotaxis receptor |
