# Supplementary material for: The Janthinobacterium sp. HH01 Genome Encodes a Homologue of the V. cholerae CqsA and L. pneumophila LqsA Autoinducer Synthases
Source: PLoS One. 2013 Feb 6;8(2):e55045. doi: 10.1371/journal.pone.0055045 (PMC3566124; doi:10.1371/journal.pone.0055045)
Supplement: Table S6 — Genes/ORFs linked to protein secretion. (DOCX) [file pone.0055045.s008.docx]

| **Secretion system** | **Locus tag** | **Gene** | **Predicted function** |
| --- | --- | --- | --- |
| **TAT** |  |  |  |
|  | Jab_1c00810 | *tatC* | sec-independent protein translocase protein TatC |
|  | Jab_1c00820 | *tatB* | sec-independent protein translocase protein TatB |
|  | Jab_1c00830 | *tatA* | sec-independent protein translocase protein TatA/E |
| **SRP** |  |  |  |
|  | Jab_1c13340 | *ftsY* | signal recognition particle-docking protein FtsY |
|  | Jab_2c29130 | *ffh* | signal recognition particle protein Ffh |
| **SEC** |  |  |  |
|  | Jab_1c05420 | *secB* | protein-export protein SecB |
|  | Jab_1c05130 | *secD1* | putative general secretion pathway protein SecD |
|  | Jab_1c06300 | *secE* | preprotein translocase SecE subunit SecE |
|  | Jab_1c07440 | *secY* | preprotein translocase subunit SecY |
|  | Jab_1c13660 | *secA* | protein translocase subunit SecA |
|  | Jab_1c21670 | *secD2* | protein translocase subunit SecD |
|  | Jab_2c17990 | *secG* | preprotein translocase subunit SecG |
|  | Jab_2c33490 | *secF* | protein-export membrane protein SecF |
|  | Jab_2c33500 | *secD3* | protein-export membrane protein SecD |
|  | Jab_2c33510 | *yajC* | preprotein translocase YajC subunit YajC |
|  | Jab_2c34390 | *oxaA* | inner membrane protein OxaA |
| **T1SS** |  |  |  |
|  | Jab_1c06010 |  | putative toxin secretion ABC transporter, ATP-binding protein |
|  | Jab_1c06020 |  | putative secretion protein |
|  | Jab_1c07640 |  | putative HlyD family secretion protein |
|  | Jab_2c01360 |  | secretion protein HlyD family |
|  | Jab_2c26300 |  | secretion protein HlyD family |
|  | Jab_2c27170 | *tolC1* | type I secretion outer membrane protein TolC |
|  | Jab_2c27180 | *hlyD* | type I secretion membrane fusion protein HlyD |
|  | Jab_2c27190 | *prtD* | type I secretion system ATPase PrtD |
|  | Jab_2c31770 | *tolC2* | type I secretion outer membrane protein TolC |
|  | Jab_2c35110 |  | type I secretion membrane fusion protein, HlyD family |
|  | Jab_2c35120 | *cyaB* | cyclolysin secretion/processing ATP-binding protein CyaB |
| **T2SS** |  |  |  |
|  | Jab_1c04300 | *pulD* | type II secretory pathway, component PulD |
|  | Jab_1c05160 |  | type II secretion system protein E |
|  | Jab_1c05170 |  | type II secretion system protein |
|  | Jab_1c05180 |  | type II secretion system protein |
|  | Jab_1c05500 | *gspG1* | general secretion pathway protein G |
|  | Jab_1c05510 | *gspH* | general secretion pathway protein H |
|  | Jab_1c05520 | *gspI* | general secretion pathway protein I |
|  | Jab_1c05530 | *gspJ* | general secretion pathway protein J |
|  | Jab_1c05540 | *gspK* | general secretion pathway protein K |
|  | Jab_1c05550 | *gspL* | general secretion pathway protein L |
|  | Jab_1c05560 | *gspM* | general secretion pathway protein M |
|  | Jab_1c05570 | *gspN* | general secretion pathway protein N |
|  | Jab_1c05580 | *gspD* | general secretion pathway protein D |
|  | Jab_1c05590 | *gspE* | general secretion pathway protein E |
|  | Jab_1c05600 | *gspF* | general secretion pathway protein F |
|  | Jab_1c17280 | *exeA* | general secretion pathway protein A |
|  | Jab_1c19410 | *gspG2* | general secretion pathway protein G |
|  | Jab_1c19420 |  | type II secretion system protein F |
|  | Jab_1c19430 |  | type II secretion system protein E |
|  | Jab_1c19480 |  | Gspd-like protein |
|  | Jab_1c19490 |  | bacterial general secretion pathway protein G domain-containing protein |
|  | Jab_1c19500 |  | bacterial type II secretion system protein G domain-containing protein |
|  | Jab_1c22630 |  | type II secretion system protein E domain-containing protein |
|  | Jab_2c02590 | *xcpR* | general secretion pathway protein E |
|  | Jab_2c02610 | *exeD* | general secretion pathway protein D |
|  | Jab_2c02620 | *exeA1* | general secretion pathway protein A |
|  | Jab_2c02640 | *epsE* | general secretion pathway protein E |
|  | Jab_2c02770 |  | putative general secretion pathway protein D |
|  | Jab_2c02780 | *exeA2* | general secretion pathway protein A |
|  | Jab_2c21430 |  | type II secretion system protein |
|  | Jab_2c25000 | *gspE1* | general secretion pathway protein E |
|  | Jab_2c29590 | *gspF* | general secretion pathway protein F |
|  | Jab_2c29600 | *gspE2* | general secretion pathway protein E |
|  | Jab_2c29660 | *gspD* | general secretion pathway protein D |
|  | Jab_2c29670 | *gspG3* | general secretion pathway protein G |
|  | Jab_2c29680 | *gspG4* | general secretion pathway protein G |
| **T6SS** |  |  |  |
|  | Jab_1c00420 |  | type VI secretion system, Rhs element Vgr family |
|  | Jab_1c15640 |  | type VI secretion protein |
|  | Jab_1c15650 |  | type VI secretion protein |
|  | Jab_1c15660 |  | virulence factor secretion apparatus protein |
|  | Jab_1c15670 |  | type VI secretion protein, lysozyme-like protein |
|  | Jab_1c15680 |  | type VI secretion protein |
|  | Jab_1c15690 |  | type VI secretion protein |
|  | Jab_1c15700 | *clpV* | type VI secretion ATPase ClpV |
|  | Jab_1c15710 |  | type VI secretion system Vgr family protein |
|  | Jab_1c15830 |  | type VI secretion protein ImpA family |
|  | Jab_2c10240 | *clpV11* | protein ClpV1 |
|  | Jab_2c10260 |  | putative type VI secretion protein, VC_A0110 family |
|  | Jab_2c10270 |  | type VI secretion system lysozyme-like protein |
|  | Jab_2c19030 | *impA* | type VI secretion protein ImpA |
|  | Jab_2c19040 | *clpV12* | type VI secretion system ATPase ClpV1 |
|  | Jab_2c19050 | *hsiF* | type VI secretion system lysozyme-like family protein HsiF |
|  | Jab_2c19060 | *hcp1* | type VI secretion system effector Hcp1 family |
|  | Jab_2c19070 | *hsiC1* | type VI secretion protein, ImpA family |
|  | Jab_2c19080 | *hsiB1* | type VI secretion system protein, VC_A0107 family |
|  | Jab_2c19100 | *lip1* | type VI secretion lipoprotein VC_A0113 family |
|  | Jab_2c19110 | *hsiJ1* | type VI secretion system protein, VC_A0114 family |
|  | Jab_2c19120 | *dotU1* | type VI secretion system inner membrane protein DotU family |
|  | Jab_2c19130 |  | OmpA/MotB domain containing protein |
|  | Jab_2c19140 |  | type VI secretion system protein, BMA_A0400 family |
|  | Jab_2c19150 | *icmF1* | type VI secretion system protein IcmF |
