# Supplementary material for: The Janthinobacterium sp. HH01 Genome Encodes a Homologue of the V. cholerae CqsA and L. pneumophila LqsA Autoinducer Synthases
Source: PLoS One. 2013 Feb 6;8(2):e55045. doi: 10.1371/journal.pone.0055045 (PMC3566124; doi:10.1371/journal.pone.0055045)
Supplement: Table S7 — Secondary metabolite gene clusters in HH01. NRPS (non-ribosomal peptide synthetases) and PKS (polyketide synthase) proteins are shown in bold. Adenylation (A) with specificity determined by NRPS predictor 2, thiolation (T), condensation (C), condensation/epimerization (C/E), epimerization (E), Coenzyme A ligase (CAL), methyltransferase (MT), thioesterase (TE), reduction (RED), ketosynthase (KS), acyltransferase (AT), ketoreductase (KR). (DOCX) [file pone.0055045.s009.docx]

| **Cluster** | **Locus tag** | **Predicted function** |
| --- | --- | --- |
| **Cluster 1** | Jab_1c00030 | hypothetical protein |
|  | Jab_1c00040 | probable branched-chain-amino-acid |
|  | Jab_1c00050 | hypothetical protein |
|  | Jab_1c00060 | putative secreted protein |
|  | Jab_1c00070 | single-stranded DNA-binding protein |
| **Cluster 2** | Jab_1c25730 | Aspartokinase |
|  | Jab_1c25770 | Peptidase M61 domain protein |
|  | Jab_1c25780 | Diaminobutyrate-2-oxoglutarate transaminase |
|  | Jab_1c25790 | ATP-binding protein SyrD |
|  | Jab_1c25800 | linear gramicidin dehydrogenase LgrE |
|  | Jab_1c25810 | MbtH domain-containing protein |
|  | Jab_1c25820 | NRPS (CAL C A_leu_ T C A_glu_ T C A_glu_ T C A_glu_ T C A_glu_ T C A_aad_ T) |
|  | Jab_1c25830 | NRPS (C A_gly_ T C A_gly_ T C) |
|  | Jab_1c25840 | para-aminobenzoate synthase Pab |
|  | Jab_1c25850 | NRPS (A_???_) |
|  | Jab_1c25860 | NRPS fragment (T C A_gly_) |
| **Cluster 3** | Jab_2c00020 | NRPS (A_glu_ T C/E A_val_ T C/E A_glu_ T C/E A_val_ T C/E A_ser_ T C/E A_gly_ T C) |
|  | Jab_2c00030 | NRPS (A_ser_ T C/E A_glu_ T C/E A_cys_ T) |
|  | Jab_2c00040 | NRPS (C) |
|  | Jab_2c00050 | NRPS (C A_asn_ T C A_ile_ MT T C A_glu_ T C A_leu_ T) |
|  | Jab_2c00060 | NRPS (C A_ser_ T C A_leu_ T C/E A_phe_ T C A_ser_ T C A_phe_ T TE) |
|  | Jab_2c00070 | putative 4'-phosphopantetheinyl tranferase |
| **Cluster 4 and Cluster 5** | Jab_2c00820 | UPF0142 protein ycaO |
|  | Jab_2c07140 | Beta-lactamase |
|  | Jab_2c07150 | hypothetical protein |
|  | Jab_2c07160 | hypothetical protein |
|  | Jab_2c07170 | Acyl-CoA dehydrogenase |
|  | Jab_2c07180 | Acyl-CoA dehydrogenase |
|  | Jab_2c07190 | NRPS (A_tyr_ T) |
|  | Jab_2c07200 | NRPS (C A_phe_ T C A_his_ T) |
|  | Jab_2c07210 | 2,4-dichlorophenol 6-monooxygenase TfdB |
|  | Jab_2c07220 | major facilitator superfamily MFS 1 |
|  | Jab_2c07230 | NRPS (C A_glu_ T C/E A_bht_ MT T C A_ala_ T E) |
|  | Jab_2c07240 | PKS-NRPS (KS AT_mal_ KR T C A_phe_ T) |
|  | Jab_2c07250 | Integrase family protein |
|  | Jab_2c07260 | hypothetical protein |
|  | Jab_2c07270 | signal transduction histidine kinase with CheB and CheR activity |
|  | Jab_2c07280 | ATP-binding protein SyrD |
|  | Jab_4c07010 | hypothetical protein |
|  | Jab_2c07290 | Ferric iron reductase protein FhuF |
|  | Jab_2c07300 | Acyl-homoserine lactone acylase pvdQ |
|  | Jab_2c07310 | Pyoverdine synthetase F |
|  | Jab_2c07320 | L-ornithine 5-monooxygenase PvdA |
|  | Jab_2c07330 | Ferrichrysobactin receptor |
|  | Jab_2c07340 | NRPS (C/E A_gly_ T TE) |
|  | Jab_2c07350 | NRPS (C A_thr_ T C/E A_lys_ T C A_ser_ T) |
|  | Jab_2c07360 | NRPS (C A_ser_ T C/E A_cys_ T C/E A_thr_ T C A_thr_ T) |
|  | Jab_2c07370 | NRPS (C A_asp_ T E C A_thr_ T) |
|  | Jab_2c07380 | NRPS (CAL T C A_ser_ T) |
|  | Jab_2c07390 | Taurine catabolism dioxygenase TauD, TfdA family |
|  | Jab_2c07400 | 4'-phosphopantetheinyl transferase superfamily |
|  | Jab_2c07410 | Thioesterase domain protein |
|  | Jab_4c07150 | hypothetical protein |
|  | Jab_2c07420 | MbtH domain containing protein |
|  | Jab_2c07430 | hypothetical protein |
|  | Jab_2c07440 | hypothetical protein |
|  | Jab_2c07450 | RNA polymerase sigma factor, sigma-70 family |
|  | Jab_2c07460 | hypothetical protein |
|  | Jab_2c07470 | PAS/PAC sensor hybrid histidine kinase |
|  | Jab_2c07480 | hypothetical protein |
|  | Jab_2c07490 | hypothetical protein |
|  | Jab_2c07500 | putative monooxygenase, FAD-binding |
| **Cluster 6** | Jab_2c09040 | 2-hydroxy-3-oxopropionate reductase GlxR |
|  | Jab_2c09050 | Hydroxypyruvate isomeraseHyi |
|  | Jab_2c09060 | Glyoxylate carboligase Gcl |
|  | Jab_2c09070 | HTH-type transcriptional regulator DmlR |
|  | Jab_2c09080 | NRPS (A_val_ T C A_val_ T) |
|  | Jab_2c09090 | NRPS (C A_thr_ T) |
|  | Jab_2c09100 | NRPS (C A_phe_ T RED) |
|  | Jab_2c09110 | major facilitator superfamily MFS 1 |
|  | Jab_2c09120 | hypothetical protein |
|  | Jab_2c09130 | Kynurenine 3-monooxygenase |
|  | Jab_2c09140 | hypothetical protein |
|  | Jab_2c09150 | hypothetical protein |
|  | Jab_2c09160 | hypothetical protein |
|  | Jab_2c09170 | hypothetical protein |
|  | Jab_2c09180 | putative Glycosyl transferase family 2 |
|  | Jab_2c09190 | hypothetical protein |
|  | Jab_2c09200 | Phosphoribulokinase |
|  | Jab_2c09210 | putative haloacid dehalogenase-like hydrolase |
|  | Jab_2c09220 | putative class II Aldolase/Adducin |
|  | Jab_2c09230 | hypothetical protein |
|  | Jab_2c09240 | hypothetical protein |
| **Cluster 7** | Jab_2c14280 | hypothetical protein |
|  | Jab_2c14290 | Dihydroxy-acid dehydratase IlvD |
|  | Jab_2c14300 | Aldose-1-epimerase Mro |
|  | Jab_2c14310 | Alpha-N-arabinofuranosidase 2 |
|  | Jab_2c14320 | Inner membrane symporter VicJ |
|  | Jab_2c14330 | TonB-dependent receptor family protein |
|  | Jab_2c14340 | AMP nucleosidase |
|  | Jab_2c14350 | NRPS (C) |
|  | Jab_2c14360 | 4'-phosphopantetheinyl transferase |
|  | Jab_2c14370 | hypothetical protein |
|  | Jab_2c14380 | Beta-lactamase BlaA |
|  | Jab_2c14390 | Pentachlorophenol 4-monooxygenase PcpB |
| **Cluster 8** | Jab_2c35220 | hypothetical protein |
|  | Jab_2c35230 | hypothetical protein |
|  | Jab_2c35240 | Cation efflux system protein CzcA |
|  | Jab_2c35250 | Cation efflux system protein CzcB |
|  | Jab_2c35260 | Cobalt-zinc-cadmium outer membrane resistance protein |
|  | Jab_2c35270 | hypothetical protein |
|  | Jab_2c35280 | hypothetical protein |
|  | Jab_2c35290 | ABC-type amino acid transport periplasmic component |
|  | Jab_2c35300 | Multi sensor signal transduction histidine kinase |
|  | Jab_4c34000 | hypothetical protein |
|  | Jab_2c35310 | Isochorismatase hydrolase |
|  | Jab_2c35320 | hypothetical protein |
|  | Jab_2c35330 | hypothetical protein |
|  | Jab_2c35340 | MATE efflux family protein |
|  | Jab_2c35350 | hypothetical protein |
|  | Jab_2c35360 | NRPS (C) |
| **Cluster 9,**  **Violacein biosynthesis cluster** | Jab_2c08810 | L-tryptophan oxidase VioA |
|  | Jab_2c08820 | violacein biosynthesis protein VioB |
|  | Jab_2c08830 | monooxygenase VioC |
|  | Jab_2c08840 | tryptophan hydroxylase VioD |
|  | Jab_2c08850 | violacein biosynthesis protein VioE |
