# Supplementary material for: The Janthinobacterium sp. HH01 Genome Encodes a Homologue of the V. cholerae CqsA and L. pneumophila LqsA Autoinducer Synthases
Source: PLoS One. 2013 Feb 6;8(2):e55045. doi: 10.1371/journal.pone.0055045 (PMC3566124; doi:10.1371/journal.pone.0055045)
Supplement: Table S8 — HH01 genes possibly linked to cell-cell communication regulatory circuits. (DOCX) [file pone.0055045.s010.docx]

| **Locus tag** | **Gene** | **Predicted function** |
| --- | --- | --- |
| Jab_2c24330 | *jqsA* | CAI-1/LAI-1 like autoinducer synthase, JqsA |
| Jab_2c24340 | *jqsS* | CAI-1/LAI-1-like autoinducer sensor kinase/phosphatase, JqsS |
| Jab_2c24350 | *jqsR* | two-component response regulator, JqsR |
| Jab_2c08870 | *luxQ2* | autoinducer 2 sensor kinase/phosphatase, LuxQ2 |
| Jab_2c05020 | *luxQ1* | autoinducer 2 sensor kinase/phosphatase, LuxQ1 |
